# Variecolactone, a Natural PDE4 Inhibitor from Marine-Derived *Talaromyces* sp. ZSD-1, Alleviates Amyloid-β Accumulation and mtDNA Dyshomeostasis via cAMP-PKA-CREB Signaling Pathway

**DOI:** 10.3390/biom16040570

**Published:** 2026-04-12

**Authors:** Tingting Fu, Yujia Shi, Zhonglin Yang, Juan Zhou, Ling Huang, Ying Fu, Wandi Xiong

**Affiliations:** Key Laboratory of Tropical Biological Resources of Ministry of Education, School of Pharmaceutical Sciences, Hainan University, Haikou 570225, China; hy0211065@muhn.edu.cn (T.F.); 23221055000018@hainanu.edu.cn (Y.S.); 20223000588@hainanu.edu.cn (Z.Y.); juanzhou@hainanu.edu.cn (J.Z.); linghuang@hainanu.edu.cn (L.H.)

**Keywords:** variecolactone, Alzheimer’s disease, PDE4 inhibitor, amyloid-β, mitochondrial function

## Abstract

Alzheimer’s disease (AD) is characterized by amyloid-β deposition, neuroinflammation, and mitochondrial dysfunction. Phosphodiesterase 4 (PDE4), a key regulator of cyclic nucleotides in neurons, represents a promising therapeutic target for AD. In this study, we performed a PDE4 inhibition-guided screen of an in-house marine natural product library derived from marine fungi, leading to the identification of a sesterterpenoid variecolactone (VLT) as a potent PDE4 inhibitor. VLT exhibited selective PDE4D inhibition (IC_50_ = 2.302 μM) with minimal activity against other PDE subtypes. Further mechanical investigation revealed that VLT treatment elevated cAMP and p-CREB levels, reduced amyloid-β (Aβ) accumulation, promoted synaptic function, and ameliorated mitochondrial fragmentation, along with mtDNA homeostasis in the AD cell model. Moreover, under conditions of mtDNA depletion or Drp1 overexpression, VLT exerted neuroprotective effects and maintained mtDNA homeostasis via the cAMP-PKA-CREB signaling pathway. These results demonstrate that PDE4 inhibition by VLT represents a promising therapeutic strategy for AD and related neurodegenerative disorders.

## 1. Introduction

Alzheimer’s disease (AD), the most common form of dementia, poses a major and growing medical and social challenge due to its increasing prevalence in aging populations worldwide. This multifactorial neurodegenerative disease is characterized by progressive cognitive decline, logical reasoning decline, and language function impairment [1]. The main pathological features of AD include Aβ accumulation in extracellular, hyperphosphorylated tau to form neurofibrillary tangles, the degeneration of basal forebrain cholinergic neurons, oxidative stress, neuroinflammation, and mitochondrial dysfunction. These pathological processes collectively disrupt neuronal and synaptic structures and functions, finally leading to neurodegeneration. Despite decades of research, the precise etiology and pathogenesis of AD remain incompletely understood [2], underlining the urgent need to elucidate its mechanisms and identify novel therapeutic targets for the effective intervention of AD.

Amyloid plaques, as the major neuropathological hallmark of AD, are mainly composed of aggregated Aβ, which is produced from the splitting fragment of the amyloid precursor protein (APP) after processing by α-secretase, β-secretase, and γ-secretase. According to the amyloid cascade hypothesis, Aβ is thought to play a major etiological role in the progress of AD pathogenesis [3]. The Aβ accumulation exhibits neurotoxic effects that include promoting oxygen stress, disrupting calcium homeostasis, and impairing mitochondrial homeostasis. Multiple neuronal activities in the central nervous system demand a great deal of energy. Mitochondria are the major energy source pool that is responsible for supplying enough ATP to maintain neuronal homeostasis and synaptic function. Emerging evidence indicates that mitochondrial dysfunction in AD, including increased oxidation damage, defective mitochondrial biogenesis, impaired mitochondrial dynamics, increased mtDNA damage and mutations, and reduced mtDNA copies, could contribute to an early and prominent feature prior to the emergence of Aβ accumulation and the tau pathology of AD [4]. These mitochondrial abnormalities finally result in mitochondrial dyshomeostasis in the progression and pathogenesis of AD.

Mitochondria comprise a multicopy mtDNA that codes for 13 proteins, 2 ribosomal RNAs, and 22 transfer RNAs, which constitute part of the oxidative phosphorylation machinery subunits [5]. The mtDNA is highly vulnerable to oxidative stress and ROS damage due to its lack of DNA-protective histones and DNA repair mechanisms. Studies have shown that mtDNA experiences approximately 10-fold higher levels of oxidative damage than nuclear DNA in AD patients’ brains [6]. A portion of patients with accumulated mtDNA mutations exhibit pronounced cognitive impairment, which is commonly seen in AD patients, indicating the important role of mtDNA in the regulation of cognitive function [7,8]. Mitochondria exist in the cytoplasm as elaborate networks undergoing antagonistic processes of fission and fusion, which is beneficial to maintain mtDNA homeostasis and mitochondrial structural integrity. Mitochondria fission produces smaller fragmented bodies as daughter cells, which appropriately maintain mitochondrial function and mitochondrial genetic inheritance [9]. Mitochondrial fission is primarily driven by dynamin-related protein 1 (Drp1) by undergoing post-translational modifications of phosphorylation and ubiquitination [10]. The phosphorylation of Drp1 occurs at serine sites of Ser637 and Ser616. The Drp1 Ser637 phosphorylation is regulated by PKA signaling and results in inhibiting mitochondrial fission and fragmentation [11].

PDEs are responsible for the hydrolysis of cAMP/cGMP. There are 11 known subfamilies, and more than 50 isoforms of PDEs have been identified. The PDE4 subfamily is widely distributed in neuron and glial cells in the central nervous system. Inhibition of PDE4 activates the cAMP-PKA-CREB signal transition system by increasing the intracellular cAMP levels and activating the PKA-associated phosphorylation of CREB in neurons and glial cells. Substantial studies suggest that PDE4s are underexploited therapeutic targets of AD for their action on the cAMP/PKA/CREB signaling pathway [12]. The PDE4A, B, and D subtypes are mainly expressed in the brain. PDE4B is responsible for the regulation of the responses of monocytic cells under inflammatory stress, while PDE4D is pivotal for modulating the cAMP/PKA/CREB pathway for memory consolidation [13,14]. Due to the PDE4 inhibitors inducing PKA-activated phosphorylation, the upregulation of cAMP-PKA signaling could activate the phosphorylation of Drp1^S637^, inhibit mitochondrial fission and fragmentation, and maintain mtDNA homeostasis, which may be a potential therapeutic target and regulatory mechanism for improving neuronal function and cognitive impairment in AD. Despite decades of efforts in drug development for AD, the current therapies offer limited clinical benefit due to the off-target effects or hepatotoxicity issues and side effects [15,16].

Marine natural products serve as a vital source for novel drug development. The unique ecological environment of the ocean fosters remarkable biodiversity, leading to the production of natural substances with diverse chemical structures and biological activities, thereby constituting a significant “blue drug repository” [17,18]. Currently, natural products derived from marine microorganisms, particularly marine fungi, have become a crucial source of bioactive lead molecules [19,20,21,22,23,24].

To identify novel PDE4 inhibitors with potential anti-AD activity, we performed a PDE4 inhibition-guided screen of an in-house marine natural product library derived from marine fungi. This screening identified nine compounds with PDE4 inhibitory activity ranging from 14.3% to 92.7% at 10 µM. Among them, a known sesterterpenoid variecolactone (VLT, **1**) showed strong PDE4 inhibitory activity (92.7%), comparable to the positive control rolipram; however, its effects on AD pathogenesis remain unclear. In the present study, we reported the isolation, structure identification, bioactivity evaluation, and mechanistic investigation of VLT as a PDE4 inhibitor. In addition, lentivirus-mediated stable cell lines and primary cultured hippocampal neurons were established to investigate the neuroprotective effects of VLT and its regulatory mechanism on mtDNA homeostasis. Taken together, this work identifies VLT as a marine-derived sesterterpenoid PDE4 inhibitor and delineates its regulatory mechanism relevant to AD.

## 2. Materials and Methods

### 2.1. General Experimental Procedures

High-resolution mass spectrometry (HRESIMS) data were acquired on a UPLC-MS system (Agilent 6546 LC/Q-TOF, Ontario, CA, USA). All 1D and 2D NMR spectra were recorded on a Bruker AV-400 MHz NMR spectrometer (Bruker Biospin AG, Fällanden, Switzerland). Preparative HPLC was performed on a CXTH P3000 pump system equipped with a YMC-Pack ODS-AQ column (250 × 20 mm, 5 μm, 12 nm) and a UV3000 detector (CXTH Ltd., Beijing, China). For column chromatography, silica gel (100–200 mesh, Qingdao Marine Chemical Ltd., Qingdao, China) and Chromatorex C_18_ (20–45 μm, Fuji Silysia Chemical Ltd., Osaska, Japan) were employed as stationary phases.

### 2.2. Construction of an In-House Marine Natural Product Library

To access chemically diverse secondary metabolites for drug discovery, an in-house marine natural product library was constructed from two marine-derived fungal strains: *Talaromyces* sp. ZSD-21 (GenBank accession no. PX806209) and *Phoma* sp. DXH009 (GenBank accession no. PQ056952). Each strain was subjected to large-scale fermentation under optimized conditions to produce secondary metabolites. The fermented material was exhaustively extracted with organic solvents, and the resulting crude extracts were fractionated and purified through sequential chromatographic techniques, including silica gel column chromatography, reversed-phase HPLC, and preparative TLC. This process yielded a total of 66 pure compounds. The structures of all isolated compounds were unambiguously elucidated by comprehensive spectroscopic analysis, including 1D and 2D NMR spectroscopy and high-resolution mass spectrometry (HRESIMS). These 66 characterized compounds, which belong to diverse structural classes such as polyketides, terpenoids (sesterterpenoids and diterpenoids), alkaloids, anthraquinoxes, xanthones, and benzofurans, are all known secondary metabolites identified by comparison with reported data [25,26,27,28,29,30,31,32,33]. They collectively constitute our in-house marine natural product library.

### 2.3. Isolation of the PDE4 Inhibitory Compounds Screening from the Library

The fungal strain *Talaromyces* sp. ZSD-21 was retrieved from −80 °C storage and cultured on potato dextrose agar (PDA) at 28 °C for 3 days. Agar blocks containing mycelia were then transferred into seven 1 L Erlenmeyer flasks, each containing 400 mL of potato dextrose broth (PDB; 20% potato, 2% glucose, and 0.3% sea salt). Seed cultures were prepared by incubating the flasks on a rotary shaker (120 rpm) at 28 °C for 3 days. For large-scale fermentation, two hundred 500 mL cylindrical wide-mouth bottles containing a solid rice medium (120 g rice, 90 mL distilled water with 0.3% sea salt per bottle, sterilized at 121 °C for 21 min) were each inoculated with 10 mL of seed culture and incubated statically at 28 °C for 30 days.

The fermented material was exhaustively extracted with ethyl acetate (3 × 2 days, room temperature). The combined extracts were filtered and concentrated under reduced pressure to obtain a crude extract. The crude material was further partitioned between petroleum ether and ethyl acetate. The ethyl acetate-soluble portion was concentrated and then fractionated by silica gel (100–200 mesh) column chromatography using a gradient elution of dichloromethane/methanol (100:0 to 1:1, *v*/*v*), yielding 11 primary fractions (Fr.1–Fr.11).

Fraction Fr.3 (12 g) was initially dissolved in methanol, yielding a precipitate identified as compound **7** (5 g). The soluble portion was separated by reversed-phase column chromatography (20–100% methanol in water) to give 26 subfractions (Fr.3-1–Fr.3-26). Subfraction Fr.3-19 was purified by preparative HPLC (acetonitrile/water, 70:30, *v*/*v*) to afford Fr.3–19-3, which was further purified by preparative TLC (dichloromethane/methanol, 40:1) to yield compound **1** (15 mg). Subtraction Fr.3-16-1 was purified by recycling preparative HPLC recycled acetonitrile/water (ReMeCN/H_2_O, 45:55, *v*/*v*) to yield compound **2** (4.6 mg, *t*_R_ = 125 min). Subtraction Fr.3-14 was separated by preparative HPLC using (ReMeCN/H_2_O, 50:50, *v*/*v*) to yield compound **6** (20.6 mg, *t*_R_ = 52 min).

Fraction Fr.6 (7 g) was separated by reversed-phase silica gel column chromatography (methanol/water, 10:90 to 100:0, *v*/*v*, containing 0.1% TFA), yielding 37 subfractions (Fr.6-1–Fr.6-37). Subtraction Fr.6-10 was purified by recycling preparative HPLC (ReMeCN/H_2_O, 25:75, 0.1% TFA) to yield compound **4** (13 mg, *t*_R_ = 32 min). Fr.6-14 was purified under the same system (ReMeCN/H_2_O, 30:70, 0.1% TFA) to yield compound **3** (10 mg, *t*_R_ = 38 min). Fr.6-31 was purified (ReMeCN/H_2_O, 52:48, 0.1% TFA) to yield compound **9** (8.2 mg, *t*_R_ = 13 min). Compounds **5** (5.7 mg) and **8** (19.8 mg) were obtained as precipitates from subfractions Fr.6-20 and Fr.6-33 after methanol dissolution, respectively.

### 2.4. PDE4D Inhibitory Screening Assays

The expression, purification of PDE4 isoforms, and enzymatic assays for target compounds were performed as previously described [17]. Briefly, recombinant plasmids were transformed into Escherichia coli BL21 (CodonPlus, San Diego, CA, USA) competent cells, which were cultured in LB medium at 37 °C until OD_600_ reached 0.6–0.8. Protein expression was induced with 0.1 mM isopropyl β-D-thiogalactopyranoside (IPTG) and incubated at 15 °C for 24 h. Recombinant proteins were purified via Ni-NTA (Qiagen, Germantown, MD, USA), Q column (GE Healthcare, Chicago, IL, USA), and Superdex 100 (GE Healthcare, Chicago, IL, USA) columns sequentially. Enzyme activity was determined using a scintillation proximity assay with ^3^H-cAMP (GE Healthcare Chicago, IL, USA) as substrates. The assay buffer consisted of 20 mM Tris-HCl (pH 7.5), 10 mM MgCl_2_ or 4 mM MnCl_2_, 1 mM dithiothreitol, and 10–30 nM labeled nucleotide (20,000–30,000 cpm per assay). Protein samples were diluted to 2 nM in assay buffer, and reactions were conducted at room temperature for 15 min before termination with 0.2 M ZnSO_4_. The product (^3^H-AMP) was precipitated by adding 0.2 N Ba (OH)_2_, while unreacted substrate (^3^H-cAMP) remained in the supernatant. Radioactivity in the supernatant was quantified using 2.5 mL of Ultima Gold scintillation cocktail (Fisher Scientific, Waltham, MA, USA) and a PerkinElmer 4910 liquid scintillation counter. IC_50_ values were calculated via nonlinear regression using at least eight compound concentrations.

### 2.5. Cell Culture and Treatment

Human neuroblastoma cells (SH-SY5Y), human microglial clone 3 cells (HMC3), human glioblastoma astrocytoma cells (U251) were purchased from the Procell Life Science & Technology Co., Ltd. (Procell, Wuhan, China). SH-SY5Y was cultured in MEM/F12 Medium (Procell, Wuhan, China) supplemented with 10% fetal bovine serum (FBS, Gibco, Waltham, MA, USA) and 1% penicillin/streptomycin (Sigma, St. Louis, MO, USA). HMC3 and U251 were cultured in DMEM (Gibco, Waltham, MA, USA) supplemented with 10% fetal bovine serum (FBS, Gibco, Waltham, MA, USA) and 1% penicillin/streptomycin (Sigma, St. Louis, MO, USA). Cultures were maintained at 37 °C in a humidified atmosphere of 5% CO_2_.

SH-SY5Y cells were seeded in 6-well plates. When confluence reached 30–40%, cells in good condition were infected with lentivirus (OBiO Technology Corp., Ltd., Shanghai, China). Lentivirus-packaged APPswe plasmids were used at MOI 5, 10, and 20 (adjusted by cell density) to optimize MOI, with cellular morphology monitored in real time. At 24 h, medium was replaced with complete MEM/F12 (10% FBS, 100 U/mL penicillin, 100 μg/mL streptomycin). Fluorescence was observed under fluorescence microscope at 48 and 72 h; cells were harvested for protein/RNA extraction to assess infection efficiency. Successfully infected cells were designated SH-SY5Y-APP (Swe695). We used the same procedures to obtain SH-SY5Y cells stably overexpressing DRP1, which were denoted as SH-SY5Y (OE-Drp1) cells.

Primary cultures of mouse hippocampal neurons were cultured from C57BL/6J and APP/PS1 embryos on day 16. The hippocampus was excised, minced, and enzymatically digested using Neural Tissue Dissociation Kit (130-92-628, Miltenyi, Bergisch Gladbach, NRW, Germany). The neural cells were seeded on poly-L-lysine-coated coverslips and cultured in Neurobasal medium (21103-049, Gibco, Waltham, MA, USA) containing B-27 supplement (810-1048, Gibco, Waltham, MA, USA), GlutaMAX (810-1048, Gibco, Waltham, MA, USA), and 1% penicillin–streptomycin (15140-122, Gibco, Waltham, MA, USA). Half of the culture medium was replaced every 3 days. On day 7, the primary cultured neurons were treated with VLT for 24 h.

A mitochondrial DNA (mtDNA) damage model was established in cells via treatment with rhodamine 6G (83697, Sigma, St. Louis, MO, USA). A stock solution of rhodamine 6G (R6G) was prepared by dissolving 2.5 mg of R6G powder in 1 mL of ultrapure water supplemented with 3% absolute ethanol (*v*/*v*). The mixture was allowed to dissolve completely over 2 days, then filtered through a 0.22 μm membrane filter, and aliquoted for storage. Cells were pretreated with R6G (1.25 μg/mL) for 36 h.

### 2.6. Cell Viability Assay

Healthy cells in the logarithmic growth phase were selected, fully digested, and prepared into a single-cell suspension. The cells were uniformly seeded into 96-well culture plates at a density of 5 × 10^4^ cells/well, with three replicate wells set for each group. After completion of group-specific treatments, 100 μL of fresh medium containing 10% CCK-8 reagent was added to each well under light-protected conditions, and blank control wells were also established simultaneously. The culture plates were wrapped with tin foil and incubated in a constant-temperature incubator at 37 °C for 2–3 h. Immediately after incubation, the optical density (OD) value of each well was measured at a wavelength of 450 nm using a microplate reader, and cell viability was calculated accordingly.

### 2.7. cAMP ELISA Assays

cAMP complete ELISA kit (ADI-900-163A) was obtained from Enzo Life Sciences. ELISA assay kits were used according to the manufacturer’s instructions.

### 2.8. Western Blot

Cells were treated with 2 or 10 μM SYJ-21 for 24 h. After washing with PBS, total proteins were extracted on ice using cell lysis buffer for Western and IP without inhibitors (P0013J, Beyotime, Shanghai, China) containing protease and phosphatase inhibitors. Protein concentration was quantified using the BCA assay, and samples were denatured in loading buffer at 100 °C. Equal amounts of protein were separated by SDS-PAGE and transferred to methanol-activated PVDF membranes. Block the membranes for 1.5 h using 5% skim milk powder or BSA, and membranes were incubated overnight at 4 °C with primary antibodies. Primary antibodies including CREB (1:250, ab178322, Abcam, Eugene, OR, USA), p-CREB^Ser133^ (1:250, ab32096, Abcam, Eugene, OR, USA), PKA (1:500, ab75991, Abcam, Eugene, OR, USA), sAPPα(1:100, 11088, IBL, Tokyo, Japan), sAPPβ(1:100, 10321, IBL, Tokyo, Japan), OPA1(1:500, ab157457, Abcam, Eugene, OR, USA), OPA1(1:500, ab157457, Abcam, Eugene, OR, USA), MFN2 (1:500, SC-100560, Santa Cruz Biotechnology, Dallas, TX, USA), DRP1 (1:500, ab184247, Abcam, Eugene, OR, USA), p-DRP1^Ser637^ (1:500, ab193216, Abcam, Eugene, OR, USA), p-DRP1^Ser616^ (1:500, ab31474755, Abcam, Eugene, OR, USA), COX IV (1:1000, ab33985, Abcam, Eugene, OR, USA), BDNF (1:1000, ab108319, Abcam, Eugene, OR, USA), and GAPDH (1:5000, bs-10900R, Bioss, Beijing, China) were incubated at 4 °C overnight. The HRP-conjugated secondary antibodies (1:10,000, bs-40296G-IRDye800CW, mouse; bs-40295G-IRDye800CW, Rabbit, Bioss, Beijing, China) were incubated for 1 h at room temperature. After thorough washing, protein bands were visualized using ECL kit (Beyotime, Shanghai, China) and imaged for densitometric analysis. Western blot original images can be found in Supplementary Materials.

### 2.9. Immunofluorescence Analysis

Cells were seeded evenly into 24-well plates with cell slides. At 60% confluence, cells were treated with compounds and incubated at 37 °C, 5% CO_2_ for 24 h (blank, model and treatment groups included). Medium was discarded; slides were rinsed twice with PBS, fixed with 4% PFA for 20 min, and rinsed thrice with PBS. After blocking with blocking buffer for 60 min, slides were incubated with primary antibody at 4 °C overnight. The primary antibodies included CREB (1:500, ab178322, Abcam, Eugene, OR, USA), p-CREB^s133^ (1:500, ab32096, Abcam, Eugene, OR, USA), BDNF (1:1000, ab108319, Abcam, Eugene, OR, USA), TOMM20, 4G8 (1:1000, 800108, BioLegend, San Diego, CA, USA), and 6E10 (1:1000, 803001, BioLegend San Diego, CA, USA). Then, the slides were incubated with secondary antibodies for 1 h. Following three 10 min rinses with PBS, slides were air dried in the dark, mounted with DAPI-containing medium, and inverted onto glass slides.

### 2.10. Molecular Docking

Variecolactone structure was drawn using Kingdraw (http://kingdraw.cn/indexen, accessed on 13 November 2025). Crystal structure of PDE4D (PDB: 8YLC) was accessed via the Protein Data Bank (http://www.rcsb.org/pdb/home/home.do, accessed on 13 November 2025). Molecular docking was conducted with AutoDock Vina 1.1.2, and protein–ligand optimization involved PyMOL 2.6.0 and Discovery Studio.

### 2.11. RNA Extraction and Real-Time PCR Analysis

TRIzol reagent (Invitrogen, Cat# 15596018, Carlsbad, CA, USA) was employed for the isolation of total RNA from cells, with the resultant expression data quantified on an ABI StepOne Plus real-time PCR instrument (Applied Biosystems, Carlsbad, CA, USA) following the 2^−ΔΔCt^ method. The primers used for *B2M* and *ND1* are shown as follows:

*B2M*: Forward, 5′-CTTTCTGGCTGGATTGGTATCT-3′;

Reverse, 5′-CAGAATAGGCTGCTGTTCCTAC-3′;

*ND1*: Forward, 5′-AGGCTAGAGGTGGCTAGAATAA-3′;

Reverse, 5′-TCTCACCATCGCTCTTCTACT-3′.

*B2M* was used as a loading control, and relative mRNA levels were normalized to loading control *B2M*.

### 2.12. Statistical Analysis

Statistical analyses were performed using GraphPad Prism 8.0 (GraphPad Software Inc., San Diego, CA, USA; RRID:SCR_002798). Statistical analysis with parametric variables was performed using the original experiment data (non-normalized data) unless specifically stated otherwise in the figure legends. Densitometric quantification of immunoblot bands was performed using ImageJ software 1.51j8. The mean intensity of the control group across three independent experiments was set to 1, and relative fold changes were subsequently determined relative to the control group. Data are included in data analysis and presentation and expressed as the mean ± SEM. One-way ANOVA with Dunnett’s post hoc test were used for multiple groups. Significance was set at * *p* < 0.05, ** *p* < 0.01, *** *p* < 0.001, and **** *p* < 0.0001.

## 3. Results

### 3.1. Identification of PDE4 Inhibitory Compounds from a Marine Natural Product Library

To discover novel PDE4 inhibitors with potential anti-AD activity, we screened an in-house marine natural product library comprising 66 compounds derived from marine fungi. This primary screening identified nine compounds that exhibited measurable PDE4 inhibitory activity at a concentration of 10 µM, with inhibition rates ranging from 14.3% to 92.7% (Table 1). The nine compounds, variecolactone (**1**); 1, 3, 6-Trihydroxy-7- methyl-9,10-anthracenedione (**2**); 3-*de*-*O*-methyl-sulochrin (**3**); methyl 4-hydroxyphenylacetate (**4**); 1-methyl-naphthalene-2,6-dicarboxylic acid (**5**); chrysoxanthone C (**6**); secalonic acid A (**7**); secalonic acid F (**8**); and RF-3192C (**9**), were identified by comparison with the NMR and HRMS spectroscopic data from the literature (Supplementary Materials). Among them, compound **1** (variecolactone, VLT), a known sesterterpenoid, displayed the most potent inhibitory activity (92.7%), comparable to that of the positive control rolipram (56.0% at 10 µM). Compound **2** also showed moderate inhibitory activity (49.1%), while the remaining compounds (**3**–**9**) exhibited relatively weak inhibition (<23%).

### 3.2. VLT Exhibits Selective PDE4D Inhibitory Activity

**Figure 1:**
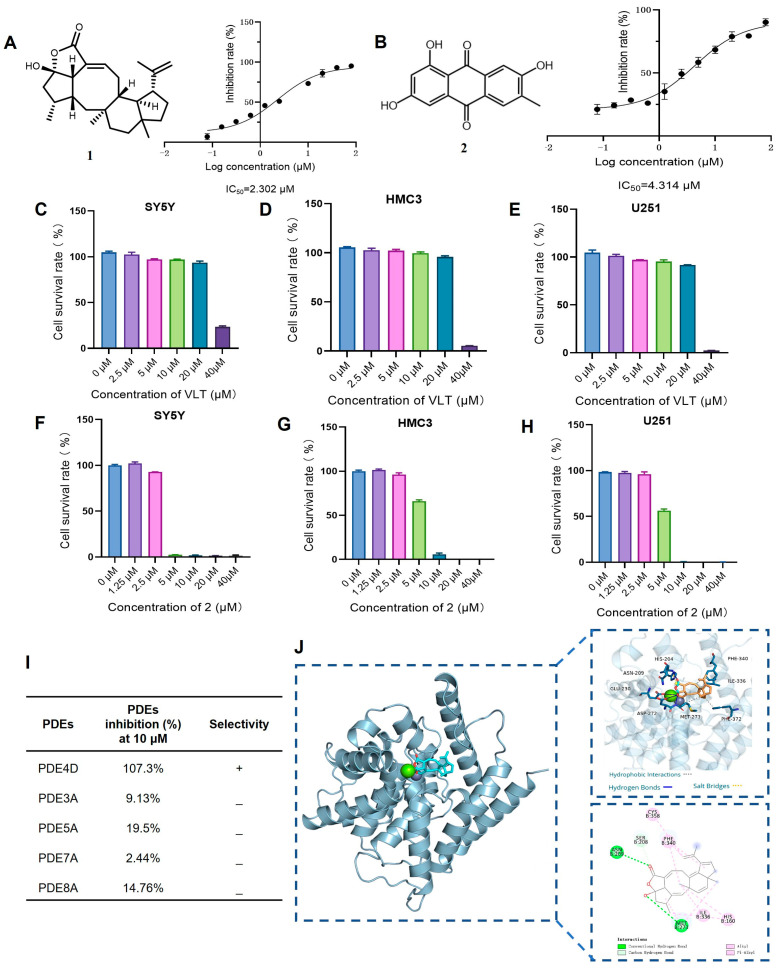
VLT exhibits preferable PDE4D inhibitory activity. (**A**,**B**) Chemical structure and plot of inhibitory effects on PDE4 by VLT and compound 2. IC_50_ values are presented as the mean ± SEM of three independent experiments. (**C**–**E**) Cell viability of SY5Y, HMC3, and U251 cells treated with different concentrations of VLT. (**F**–**H**) Cell viability of SY5Y, HMC3, and U251 cells treated with different concentrations of compound **2**. (**I**) Inhibitory activities of VLT against four PDE subtypes. (**J**) Predicted binding pattern of VLT with PDE4D (PDB: 8YLC) from molecular docking. The docking score is −8.6 ± 0.01 kcal/mol.

PDE4 plays a key role in regulating the cAMP-PKA-CREB signaling pathway and is considered as a potential target for the treatment of AD. We preliminarily evaluated inhibitory potencies of the library compounds against PDE4D, with rolipram as the positive control. As shown in Table 1, compound **1** (VLT) demonstrated the strongest inhibitory activity and was selected to test the half maximal inhibitory concentration (IC_50_), which showed an IC_50_ value of 2.302 μM, while compound **2** displayed moderate inhibitory activity against PDE4D with an IC_50_ of 4.314 μM (Figure 1A,B). VLT and **2** were further evaluated for cell viability in three human cell lines, including SY5Y, HMC3, and U251, which respectively represent neuron-like, microglia-like, and astrocyte-like cells in the brain. VLT showed cytotoxicity up to 40 μM in these three cell lines, while **2** exhibited strong cytotoxicity at a concentration of 10 μM (Figure 1C–H). In order to detect the selective specificity of the PDE4 family’s inhibitory activity of VLT, we tested the PDEs inhibitory activity of VLT on other subtypes of PDEs. As results showed, VLT only showed great inhibitory activity on PDE4D with an inhibitory ratio of 92.7%, with inhibitory ratios of 9.13% on PDE3A, 19.5% on PDE5A, 2.44% on PDE7A, and 14.76% on PDE8A, respectively (Figure 1I). In addition, molecular docking was conducted to evaluate the interactions between VLT and PDE4D. Referring to the crystallographic structure of PDE4D, VLT exhibited strong binding affinity to PDE4D, forming hydrogen bonds with Asn209, Glu230, Asp272, and Met273, and maintaining its hydrophobic interactions with active site residues Met273, Ile336, Phe340, and Phe372 in the catalytic pocket (Figure 1J). These interactions demonstrated that VLT is a selective PDE4 family inhibitor with potent inhibitory activity against PDE4D, underscoring its potential as a lead compound for AD therapy.

### 3.3. VLT Promotes Neural Function via the cAMP/CREB/BDNF Signaling Pathway

**Figure 2:**
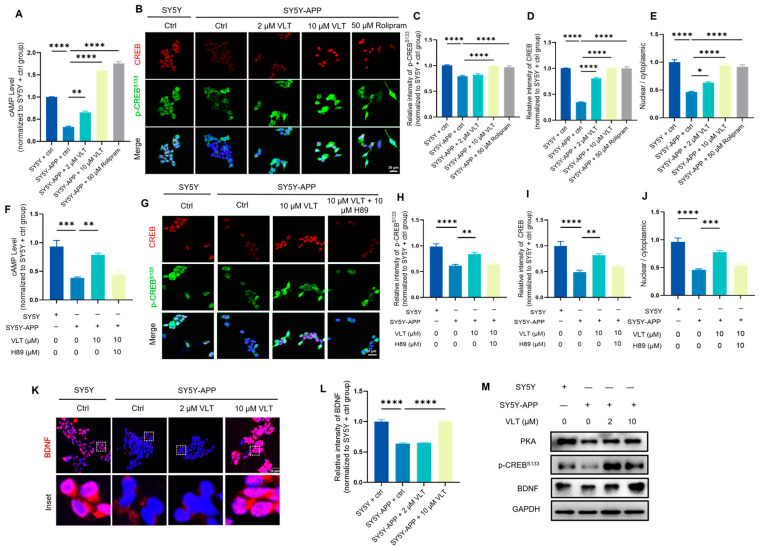
VLT promotes neural function via cAMP/PKA/CREB/BDNF signaling pathway in APP-Swe-overexpressing SH-SY5Y cells. (**A**) cAMP levels measured by competitive ELISA. A 50 μM rolipram treatment was taken as a positive control. (**B**–**D**) Representative staining images and quantification of immunofluorescence analysis for p-CREB^S133^ (green) and CREB (red). Nuclei were stained with DAPI (blue). A 50 μM rolipram treatment was taken as a positive control. Scale bar: 20 µm. (**E**) Nuclear/cytoplasmic (N:C) ratio quantified via ImageJ 1.51j8 after p-CREB^S133^ staining. (**F**) cAMP levels measured by competitive ELISA. (**G**–**I**) Representative staining images and quantification of immunofluorescence analysis for p-CREB^S133^ (green) and CREB (red). Nuclei were stained with DAPI (blue). (**J**) Nuclear/cytoplasmic (N:C) ratio quantified via ImageJ after p-CREB^S133^ staining. (**K**,**L**) Representative staining images and quantification of immunofluorescence analysis for BDNF (red). Nuclei were stained with DAPI (blue). Scale bar: 20 µm. (**M**) Protein expression levels of p-CREB^S133^, PKA, and BDNF were detected by Western blot. Data are presented as the mean ± SEM of three independent experiments. Data were analyzed by One-way ANOVA with Dunnett’s test. * *p* < 0.05, ** *p* < 0.01, *** *p* < 0.001, and **** *p* < 0.0001.

PDE4 inhibitors increase the cAMP levels and subsequently activate the cAMP/PKA/CREB signaling pathway. Phosphorylation of CREB is essential in the regulation of gene transcription of the brain-derived neurotrophic factor (BDNF), a pivotal driver of neurogenesis, synaptic plasticity, and memory formation [18]. Emerging evidence demonstrated that decreased levels of BDNF may lead to the synaptic loss and degeneration of specific neuronal populations in an AD-affected brain [18]. An increased BDNF level is associated with improved spatial memory and cognitive status, which suggests that BDNF is a possible reason for the improving neural function in AD [18,19]. To elucidate the regulatory mechanism underlying anti-AD effects of PDE4 inhibitor VLT in neural function, we established a stable APP-Swe695-overexpression SH-SY5Y cell line as an AD model cell and further illustrated how VLT contributes to AD pathogenesis. A 50 μM rolipram treatment was taken as a positive control. In line with the inhibitory activity of VLT against PDE4D in the bioactivity assay, treatment of 2 μM and 10 μM VLT efficiently restored the decreased cAMP in the AD model cell (Figure 2A). The activation of CREB and transcription and translation of BDNF rely on the phosphorylation of CREB at the Ser133 site residue. Then, we combined immunofluorescence staining of p-CREB^Ser133^ to visualize the activation of CREB and its nucleus translocation (Figure 2B). We found that VLT generally increased levels of p-CREB^Ser133^ and its nucleus translocation in the AD model cell (Figure 2C–E). To further investigate whether VLT works via the activation of the cAMP-PKA-CREB axis and targeting PDE4D, we treated the AD model cell with the PKA inhibitor H89 together with VLT and found that H89 abolished the effects of VLT on the cAMP-PKA-CREB axis (Figure 2F–M). Immunofluorescence staining results showed that the VLT treatment markedly upregulated the BDNF levels in the AD model cell (Figure 2K,L). Similarly, we performed Western blotting to detect the alteration of the PKA/CREB/BDNF axis in AD model cells. Western blot analysis revealed that VLT upregulated the phosphorylation of CREB^Ser133^, PKA, and BDNF levels (Figure 2M). These results together demonstrated that VLT improved neural function in the AD model cell via activating the cAMP/PKA/CREB/BDNF signaling pathway by inhibiting PDE4 activity.

### 3.4. Effects of VLT on Aβ Accumulation, Mitochondrial Morphology, and mtDNA Homeostasis

The downregulation of BDNF and CREB serves as two downstream mediators of Aβ toxicity. Accumulated Aβ is assumed as the main cause of neural dysfunction and cognitive impairments in AD pathogenesis. Studies have reported that AD patients’ brains and Aβ-treated neurons have reduced phosphorylation levels of CREB. The toxic effects of Aβ on neural function, synaptic plasticity, and cognitive impairment are regulated by the cAMP/PKA/CREB signaling pathway. In addition, the deleterious effects of Aβ on the BDNF could result in neuronal loss and neurodegeneration.

**Figure 3:**
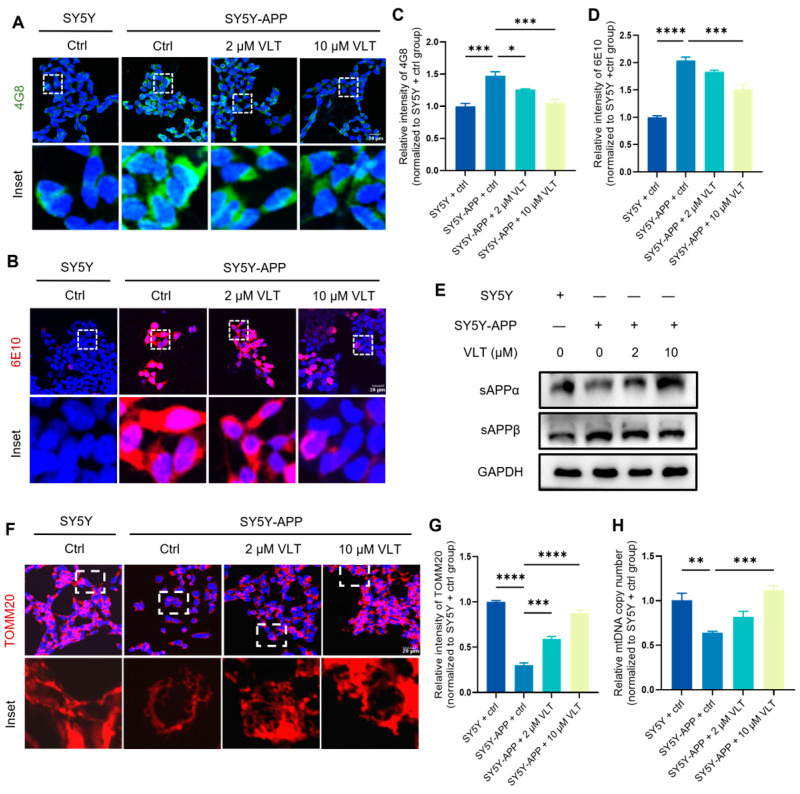
Effects of VLT on Aβ accumulation, mitochondrial morphology, and mtDNA homeostasis. (**A**–**D**) Representative staining images and quantification of immunofluorescence analysis for Aβ (stained with antibody 4G8 and 6E10). Nuclei were stained with DAPI (blue). Scale bar: 20 μm. (**E**) Protein expression levels of sAPPα and sAPPβ were detected by Western blot. (**F**,**G**) Representative staining images and quantification of immunofluorescence analysis for TOMM20 (red). Nuclei were stained with DAPI (blue). Scale bar: 20 μm. (**H**) Relative mtDNA copy number measured by qPCR (*ND1* primer, normalized to nuclear *B2M*). Untreated SY5Y cells served as 100% reference. Data are presented as the mean ± SEM of three independent experiments. Data were analyzed by One-way ANOVA with Dunnett’s test. * *p* < 0.05, ** *p* < 0.01, *** *p* < 0.001, and **** *p* < 0.0001.

Given the established link between Aβ toxicity and the suppression of the cAMP/PKA/CREB pathway, we next investigated whether VLT could mitigate Aβ pathology. 6E10 recognizes the N-terminal region of full-length Aβ and tends to detect total Aβ monomers and fibril deposits, while 4G8 exhibits a higher affinity for Aβ intermediate fragments and displays relatively better sensitivity toward oligomeric assemblies. Immunofluorescence staining with Aβ-specific antibodies 4G8 and 6E10 showed that VLT treatment significantly reduced the different aggregation states of Aβ in the AD model cell (Figure 3A–D). In order to further investigate whether the effect of VLT on Aβ accumulation is based on amyloidogenic APP processing, we used sAPPα and sAPPβ antibodies to detect APP processing intermediates. We found that VLT led to an increased level of sAPPα and a decreased level of sAPPβ in the AD model cell (Figure 3E). Although our data support a critical role of VLT in Aβ accumulation and neural dysfunction via the cAMP/PKA/CREB/BDNF axis in AD, the regulatory mechanism of VLT mediated in AD pathology remains unclear. Studies showed that Aβ has devastating effects on mitochondrial function in silico, in vitro, and in vivo [3,4,20,21]. Reciprocally, mitochondrial dysfunction also affects Aβ pathology. Due to that, mtDNA is highly responsible for the mitochondrial function; mice with overloaded mtDNA mutations showed increased Aβ deposit burden and plaque size [22,23,24]. To deeply reveal the regulatory action of VLT, we detected the alteration of mitochondrial morphology and mtDNA copy number. The immunofluorescence staining results combined with the statistical analysis of the intensity of TOMM20 demonstrated that VLT improved mitochondrial morphology, including prolonged tube-shaped mitochondria and the mitochondrial network, and decreased mitochondrial fragmentation (Figure 3E,F). Concomitantly, VLT also restored the decreased mtDNA copy number in the AD model cell (Figure 3G). Taken together, these findings indicate that VLT decreased Aβ accumulation and improved the mitochondrial structure and mtDNA homeostasis.

### 3.5. VLT Promotes Synaptic Function and Mitochondrial Dynamical Morphology in AD Primary Culture Neurons

**Figure 4:**
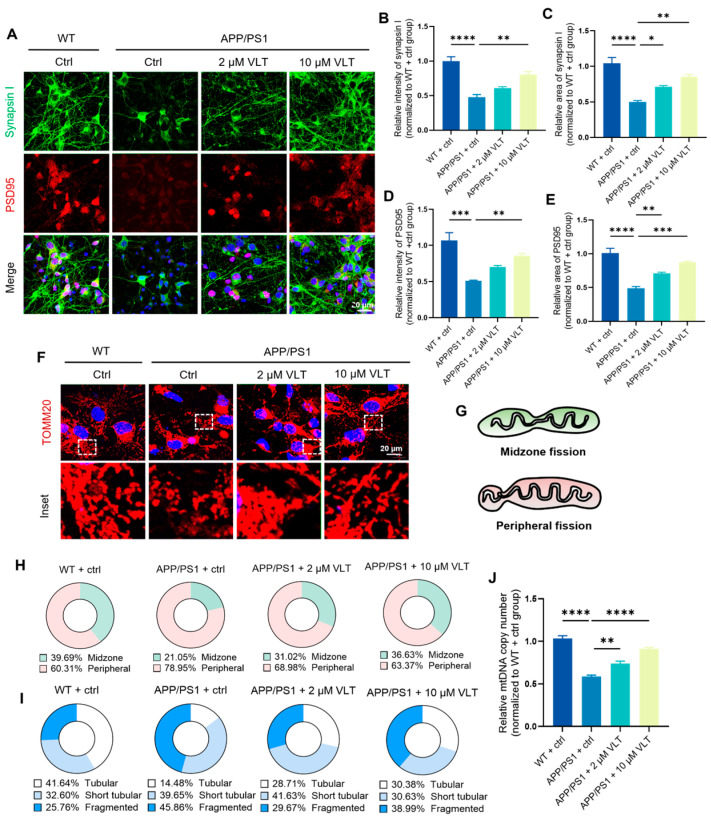
VLT promotes synaptic function and mitochondrial dynamics in AD primary culture neurons. (**A**–**E**) Representative staining images and quantification of immunofluorescence and area analysis of synapsin I (green) and PSD95 (red). Nuclei were stained with DAPI (blue). Scale bar: 20 μm. (**F**) Representative staining images of TOMM20 (red). Nuclei were stained with DAPI (blue). Scale bar: 20 μm. (**G**) Schematic diagram for midzone fission and peripheral fission of mitochondrial dynamics. (**H**) Quantification of the percentage of midzone and peripheral fission in each group. (**I**) Quantification of mitochondrial morphology in each group. (**J**) Relative mtDNA copy number measured by qPCR (*ND1* primer, normalized to nuclear *B2M*). WT cells served as 100% reference. Data are presented as the mean ± SEM of three independent experiments. Data were analyzed by One-way ANOVA with Dunnett’s test. * *p* < 0.05, ** *p* < 0.01, *** *p* < 0.001, and **** *p* < 0.0001.

Accumulated Aβ around neurons in the hippocampus could lead to synaptic dysfunction that links to cognitive impairment. To further verify the effect of VLT on synaptic function and mitochondria morphology under AD conditions, primary hippocampal neurons from APP/PS1 and a wild-type embryo were treated with VLT (0, 2, and 10 μM) at 7 days in vitro (DIV7). The treatment of 2 μM and 10 μM of VLT significantly increased the signal intensity of the postsynaptic density protein 95 (PSD95) and synapsin I in AD primary culture neurons at DIV7 (Figure 4A–E), which are crucial for the synaptic development and maturation of hippocampal neurons. The dynamic regulation of mitochondrial morphology is essential for the maintenance of neural homeostasis and synaptic energy supplementation. The immunofluorescence staining of TOMM20 demonstrated the effects of VLT on mitochondrial dynamical morphology. VLT increased the proportion of midzone fission while reduced the proportion of peripheral fission (Figure 4F–H). The increased peripheral fission events produced more fragmented mitochondria and fewer tubular mitochondria in AD primary culture neurons. These changes in mitochondrial dynamical morphology were reversed by VLT (Figure 4I). The two models of mitochondrial fission, including midzone and peripheral, could affect the downstream fate of the mitochondrial progenies and allocation of mtDNA. We found that treatment with 2 μM and 10 μM VLT could restore the decreased mtDNA levels in AD primary culture neurons at DIV7 (Figure 4J). These findings demonstrated that the novel PDE4 inhibitor VLT promoted synaptic function and mitochondrial dynamical morphology in hippocampal neurons.

### 3.6. VLT Regulates mtDNA Homeostasis via cAMP/PKA/CREB Signaling Pathway

**Figure 5:**
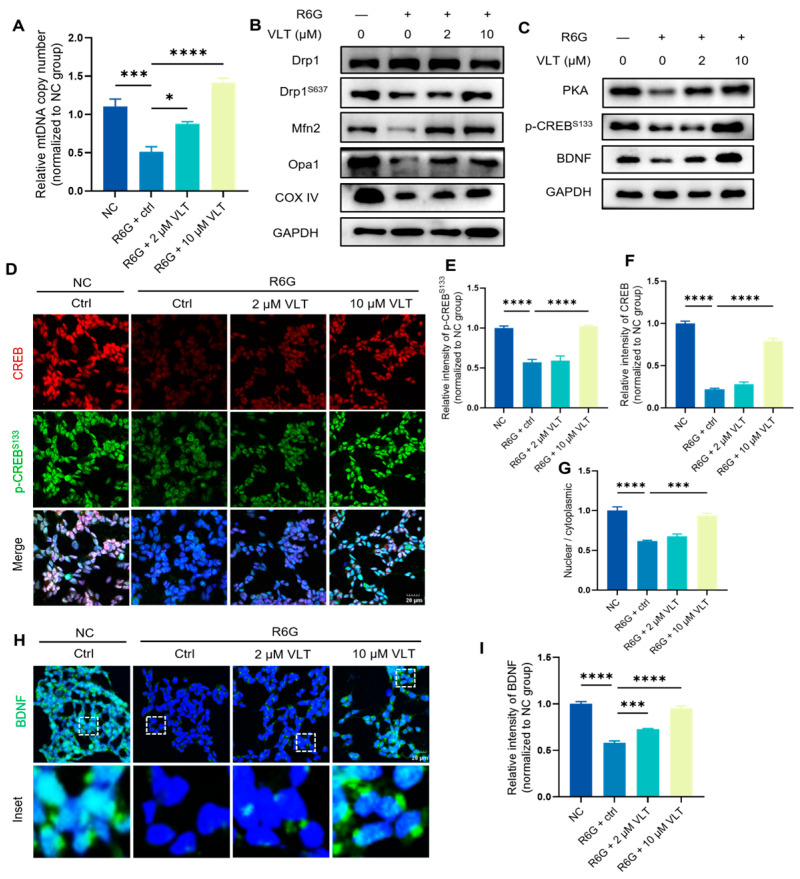
VLT rescues mtDNA homeostasis and mitochondrial dynamics via the cAMP/CREB/BDNF signaling pathway after mtDNA depletion. SH-SY5Y cells were pretreated with 1.25 μg/mL rhodamine 6G (R6G) for 36 h. (**A**) Relative mtDNA copy number measured by qPCR (*ND1* primer, normalized to nuclear *B2M*). Untreated SY5Y cells served as 100% reference. (**B**) Protein expression levels of Drp1, Drp1^S637^, Mfn2, Opa1, and COX IV were detected by Western blot. (**C**) Protein expression levels of p-CREB^S133^, PKA, and BDNF were detected by Western blot. (**D**–**F**) Representative staining images and quantification of immunofluorescence analysis for p-CREB^S133^ (green) and CREB (red). Nuclei were stained with DAPI (blue). Scale bar: 20 µm. (**G**) Nuclear/cytoplasmic (N:C) ratio quantified via ImageJ after p-CREB^S133^ staining. (**H**,**I**) Representative staining images and quantification of immunofluorescence analysis for BDNF (green). Nuclei were stained with DAPI (blue). Scale bar: 20 µm. Data are presented as the mean ± SEM of three independent experiments. Data were analyzed by One-way ANOVA with Dunnett’s test. * *p* < 0.05, *** *p* < 0.001, and **** *p* < 0.0001.

Revealing the effects mediated by VLT on mtDNA homeostasis requires a rational model. We pretreated the SH-SY5Y with 1.25 μg/mL rhodamine 6G (R6G) for 36 h, which irreversibly binds to mitochondrial oxidative phosphorylation and induces mitochondrial dysfunction. Pretreatment of R6G markedly decreased the mtDNA copy number by 50% and reduced the levels of mitochondrial dynamical proteins, including Drp1^S637^, mitofusin2 (Mfn2), and optic atrophy 1 (Opa1), while the level of Drp1 was increased (Figure 5A,B). The cytochrome c oxidase subunit IV (COX IV) represents the mitochondrial oxidative phosphorylation activity. Pretreatment of R6G also decreased the expression level of COX IV. Treatment of VLT abrogated R6G-induced mitochondrial dysfunction with improved parameters of mtDNA copy number and mitochondrial dynamic proteins (Figure 5A,B). Next, we performed Western blotting to detect the alteration of the PKA/CREB/BDNF axis after mtDNA depletion. The result showed that VLT increased the levels of p-CREB^Ser133^, PKA, and BDNF in R6G-treated cells (Figure 5C). Furthermore, immunofluorescence staining confirmed that VLT promoted both the phosphorylation and nucleus distribution of CREB^Ser133^ and increased BDNF expression after mtDNA depletion (Figure 5D–I). We found that VLT increased levels of p-CREB^Ser133^ and its nucleus translocation after mtDNA depletion (Figure 5E–G). Together, these data demonstrated that VLT improved mtDNA homeostasis and mitochondrial dynamics via the PKA/CREB/BDNF signaling pathway by inhibiting PDE4 activity after mtDNA depletion.

**Figure 6:**
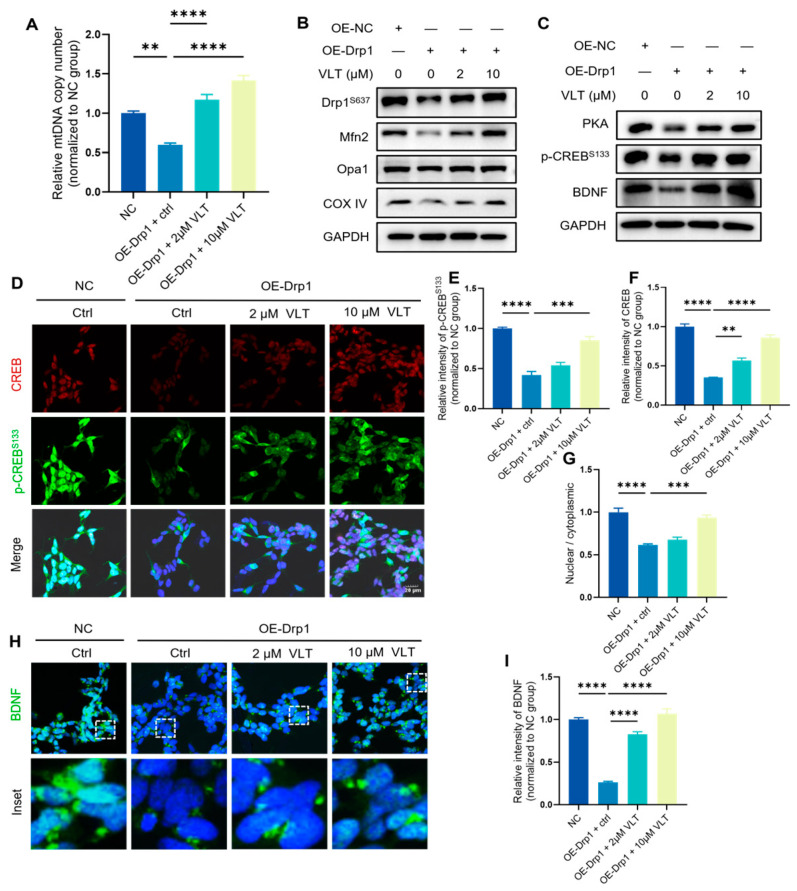
VLT regulates mtDNA homeostasis via the PKA/CREB/Drp1 signaling pathway. (**A**) Relative mtDNA copy number measured by qPCR (*ND1* primer, normalized to nuclear *B2M*) in Drp1-overexpressing SH-SY5Y cells treated with VLT. Untreated SY5Y cells served as 100% reference. (**B**) Protein expression levels of Drp1^S637^, Mfn2, Opa1, and COX IV were detected by Western blot. (**C**) Protein expression levels of p-CREB^S133^, PKA, and BDNF were detected by Western blot. (**D**–**F**) Representative staining images and quantification of immunofluorescence analysis for p-CREB^S133^ (green) and CREB (red). Nuclei were stained with DAPI (blue). Scale bar: 20 µm. (**G**) Nuclear/cytoplasmic (N:C) ratio quantified via ImageJ after p-CREB^S133^ staining. (**H**,**I**) Representative staining images and quantification of immunofluorescence analysis for BDNF (green). Nuclei were stained with DAPI (blue). Scale bar: 20 µm. Data are presented as the mean ± SEM of three independent replicates. Data were analyzed by One-way ANOVA with Dunnett’s test. ** *p* < 0.01, *** *p* < 0.001, and **** *p* < 0.0001.

To investigate how VLT regulates mtDNA homeostasis by inhibiting PDE4, we detected the levels of cAMP/PKA/Drp1 after the overexpression Drp1. Mitochondria are extremely dynamic organelles that ceaselessly experience fission and fusion. A delicate balance of mitochondria fission and fusion is essential to restore organelles’ health and homogeneity, especially to maintain mtDNA homeostasis, complementation, and genetic exchange [9,34,35,36]. Drp1, as the master mediator of mitochondrial fission, is closely associated with mitochondrial dynamic homeostasis. Excessive levels of Drp1 significantly induce mitochondrial dysfunction and trigger mtDNA stress [9]. Drp1 undergoes various post-transcriptional modifications, including phosphorylation and sumoylation. PKA-mediated phosphorylation of Drp1 could lead to mitochondrial elongation and remodeling the mitochondrial structure by reducing the proportion of mitochondrial fragmentation [9,35,37,38]. Our data demonstrated that VLT abrogated the decreased alterations of the mtDNA copy number after overexpression of Drp1 (Figure 6A). The levels of mitochondrial dynamic proteins, including Drp1^S637^, Mfn2, and Opa1, and mitochondrial oxidative phosphorylation (OXPHOS) protein COX IV significantly increased with the treatment of VLT in cells with an overexpression of Drp1 (Figure 6B). Then, we conducted Western blotting to analyze the alteration of the PKA/CREB/BDNF axis after mtDNA depletion. The result showed that treatment of VLT restored the protein levels of phosphorylated CREB^Ser133^, PKA, and BDNF levels in cells with an overexpression of Drp1 (Figure 6C). In addition, we combined immunofluorescence staining of p-CREB^Ser133^ for the visualization of the phosphorylation of CREB and its nucleus distribution (Figure 6D). We found that VLT increased levels of p-CREB^Ser133^ and its nucleus translocation after overexpression of Drp1 (Figure 6E–G). Similar to the results of Western blotting, immunofluorescence staining of BDNF showed that the treatment with VLT significantly upregulated the BDNF levels after mtDNA depletion (Figure 6H,I). These molecular biological results together demonstrated that VLT regulates mtDNA homeostasis via PKA/CREB/Drp1 signaling pathway by inhibiting PDE4 activity after the overexpression of Drp1. Thus, treatment with the PDE4 inhibitor VLT may provide a novel therapeutic for AD.

## 4. Discussion

PDEs have long been recognized as promising therapeutic targets for AD. PDE inhibitors exert prominent cognition-improving effects by elevating intracellular cAMP and cGMP levels. Accumulating evidence demonstrates that PDE4 represents a critical therapeutic target for AD, owing to its central regulation of the cAMP/PKA/CREB signaling associated with cognitive function and memory formation [39,40]. One major pharmacological strategy targeting PDE4 relies on sustained PDE4 inhibition, which persistently activates CREB phosphorylation—events essential for maintaining synaptic plasticity and consolidating memory [41,42,43]. Nevertheless, the clinical development of PDE4 inhibitors for AD treatment still faces considerable challenges, including unavoidable side effects and an incomplete understanding of the interplay between PDE4 activity and AD pathological progression. In the present study, we constructed a marine microbial compound library and performed a bioactivity screening guided by PDE4 inhibitory evaluation. The fungal strain *Talaromyces* sp. ZSD-21 was identified as a promising resource for natural PDE4 inhibitors. Through bioassay-guided isolation, nine compounds (including two newly identified metabolites) with varying PDE4 inhibitory potency were obtained. Among these isolates, the known VLT exhibited the strongest PDE4 inhibition, with an inhibition rate of 92.7% at 10 μM and an IC_50_ value of 2.302 μM. Notably, variecolin and its analogs have been reported to possess potent anticancer and anti-inflammatory activities both in vitro and in vivo [44,45]. Our findings therefore expand the biological functional profile of VLT and highlight its potential as a natural PDE4 inhibitor for anti-AD intervention.

Previous studies have demonstrated that variecolin analogs exhibit optimized pharmacological properties and a wider therapeutic window in tumor-bearing animal models [44]. However, our data indicate that VLT displays a relatively narrow safety profile in neuronal cells, suggesting that further structural modification is required to reduce its cytotoxicity and improve its neuronal tolerance. PDE subtype selectivity assays confirmed that VLT preferentially binds to the PDE4 subfamily rather than other PDE isoforms. Molecular docking analysis revealed that VLT forms stable hydrophobic interactions with Met273, Ile336, Phe340, and Phe372, while establishing hydrogen bonds with Asn209, Glu230, Asp272, and Met273 within the PDE4D active pocket. Although molecular docking provides predictive evidence for the binding mode between VLT and PDE4D, further experimental validation is still necessary to confirm its molecular interaction and functional regulation. To systematically verify the neuroprotective effects of VLT against AD pathogenesis, we established an APP_Swe_-overexpressing SH-SY5Y cell line and cultured primary hippocampal neurons as classic in vitro AD models. Functional evaluation confirmed that VLT ameliorated neuronal dysfunction and upregulated the core protein expression of the PKA/CREB/BDNF signaling pathway in AD model cells. Immunofluorescence results further demonstrated that VLT markedly reduced intracellular Aβ deposition and rescued impaired synaptic function compared with the control group. Mechanistically, VLT restored abnormal mitochondrial morphology and rescued mtDNA dyshomeostasis through activation of the cAMP/CREB/BDNF signaling axis.

Neuronal function is highly dependent on intact mitochondrial energy metabolism. Excessive Aβ accumulation directly disrupts mitochondrial homeostasis and triggers mitochondrial dysfunction, which is characterized by impaired ATP production, compromised OXPHOS activity, aberrant mitochondrial dynamics, and disrupted mtDNA stability, and finally leads to neuronal dysfunction [46,47]. Mitochondrial dynamics, governed by proteins like Drp1, play a critical role in nuclear–mitochondrial crosstalk by regulating the distribution and exchange of mtDNA and mitochondrial proteins across the mitochondrial network [48]. As a core structural component of the mitochondrial outer membrane translocase complex, TOMM20 is responsible for recognizing and importing nuclear-encoded mitochondrial proteins, especially those closely associated with mitochondrial dynamics such as Drp1. Under pathological AD conditions, decreased TOMM20 could impair the cytoplasmic import of dynamic regulatory proteins and aggravate mitochondrial fragmentation and dysfunction. Consistent with previous reports [46,47], our results revealed excessive peripheral fission and severe mitochondrial fragmentation under AD pathological conditions. We found that VLT significantly upregulated TOMM20 expression through PDE4 inhibition and further confirmed that VLT effectively restored the mtDNA dyshomeostasis induced by mtDNA depletion via the cAMP/CREB/BDNF pathway; similar protective effects were also observed in Drp1-overexpressing neuronal models. As reported, baicalin derived from *Radix scutellariae* alleviates synaptic deficits and mitochondrial fragmentation by inhibiting PDE4 and promoting Drp1 phosphorylation at Ser637 [48]. There is a critical but underemphasized aspect of mitochondrial function in AD, which is nuclear–mitochondrial crosstalk, which ensures the mtDNA homeostasis, mitochondrial biogenesis, and OXPHOS activity [46,49]. The OXPHOS activity requires the coordinated expression of mtDNA-encoded subunits and multiple nDNA-encoded subunits. Under AD pathological conditions, Aβ-induced oxidative stress disrupts nuclear–mitochondrial crosstalk by disrupting mtDNA homeostasis and the nuclear transcription of OXPHOS-related genes, further creating a vicious cycle of mitochondrial dysfunction [47]. Importantly, our study demonstrated that VLT enhanced nuclear–mitochondrial crosstalk by maintaining mtDNA homeostasis and upregulating COX IV expression via the PDE4/cAMP/PKA/CREB axis. Collectively, these findings support that the PDE4 inhibitor VLT remodels mitochondrial dynamics and stabilizes mtDNA homeostasis by regulating Drp1 phosphorylation and activating the PDE4/cAMP/PKA/Drp1 signaling cascade.

Overall, the present study still has several notable limitations that need to be acknowledged. First, although VLT exhibits a potent inhibitory effect on PDE4 with an IC_50_ of approximately 2 μM and exerts satisfactory neuroprotective activity in cellular AD models, its in vivo biosafety profile and blood–brain barrier permeability remain uncharacterized. Second, all mechanistic investigations in this work are currently restricted to the cellular level. Therefore, future research will incorporate behavioral assessments and histopathological examinations using classic AD animal models to further validate the therapeutic efficacy of VLT against AD. In addition, mainstream therapeutic strategies for AD focus on targeting upstream pathological events, Aβ, or tau aggregation. In contrast, the natural PDE4 inhibitor VLT intervenes in the downstream pathological cascades induced by Aβ aggregation, including mitochondrial dysfunction, impaired cAMP/CREB/BDNF signaling, and neuronal dysfunction. Although VLT does not target the initial protein aggregation events, such downstream intervention still holds significant clinical value: it can effectively alleviate neuronal functional impairment, relieve cognitive deficits, and slow down the progressive deterioration of AD pathology, thereby providing a feasible symptomatic and disease-modifying strategy.

## 5. Conclusions

Collectively, this study underscores that VLT emerges as a novel marine-derived sesterterpenoid PDE4 inhibitor that improves neural function, mitochondrial homeostasis, and Aβ pathology through a multimodal mechanism. These findings provide both a promising lead compound and a mechanistic framework for the development of PDE4-targeted therapies for AD.

## Figures and Tables

**Table 1:** PDE4 inhibition (%) of compounds **1**–**9** at 10 µM.

Compound No. or Name	Structure	PDE4 Inhibition	Compound No. or Name	Structure	PDE4 Inhibition
**1** (VTL)	** 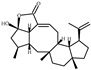 **	92.7	**6**	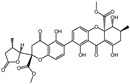	19.7
**2**	** 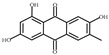 **	49.1	**7**	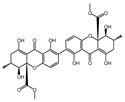	22.6
**3**	** 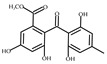 **	14.3	**8**	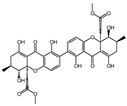	19.4
**4**	** 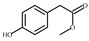 **	21.6	**9**	** 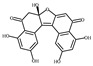 **	22.0
**5**	** 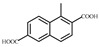 **	16.5	**Rolipram**	** 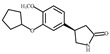 **	56.0

## Data Availability

The data presented in this study is available within the article or Supplementary Materials.

## Supplementary Materials

The following supporting information can be downloaded at https://www.mdpi.com/article/10.3390/biom16040570/s1: NMR data of compounds **1**–**9**; spectroscopic diagrams of compounds **1**–**9**. Figure S1: ^1^H NMR spectrum (Chloroform-*d*) of 1; Figure S2: ^13^C NMR spectrum (Chloroform-*d*) of 1; FigureS3: HRESIMS spectrum of 1; Figure S4: ^1^H NMR spectrum (Dimethyl Sulfoxide-*d6*) of 2; Figure S5: ^13^C NMR spectrum (Dimethyl Sulfoxide-*d6*) of 2; Figure S6: HRESIMS spectrum of 2; Figure S7: ^1^H NMR spectrum (Acetone-*d6*) of 3; Figure S8: ^13^C NMR spectrum (Acetone-*d6*) of 3; Figure S9: HRESIMS spectrum of 3; Figure S10: ^1^H NMR spectrum (Methanol-*d4*) of 4; Figure S11: ^13^C NMR spectrum (Methanol-*d4*) of 4; Figure S12: HRESIMS spectrum of 4; Figure S13: ^1^H NMR spectrum (Dimethyl Sulfoxide-*d6*) of 5; Figure S14: ^13^C NMR spectrum (Dimethyl Sulfoxide-*d6*) of 5; Figure S15: HRESIMS spectrum of 5; Figure S16: ^1^H NMR spectrum (Dimethyl Sulfoxide-*d6*) of 6; Figure S17: ^13^C NMR spectrum (Dimethyl Sulfoxide-*d6*) of 6; Figure S18: HRESIMS spectrum of 6; Figure S19: ^1^H NMR spectrum (Dimethyl Sulfoxide-*d6*) of 7; Figure S20: ^13^C NMR spectrum (Dimethyl Sulfoxide-*d6*) of 7; Figure S21: HRESIMS spectrum of 7; Figure S22: ^1^H NMR spectrum (Acetone-*d6*) of 8; Figure S23: ^13^C NMR spectrum (Acetone-*d6*) of 8; Figure S24: HRESIMS spectrum of 8; Figure S25: ^1^H NMR spectrum (Dimethyl Sulfoxide-*d6*) of 9; Figure S26: ^13^C NMR spectrum (Dimethyl Sulfoxide-*d6*) of 9; Figure S27: HRESIMS spectrum of 9.
