# Supplementary material for: Variecolactone, a Natural PDE4 Inhibitor from Marine-Derived Talaromyces sp. ZSD-1, Alleviates Amyloid-β Accumulation and mtDNA Dyshomeostasis via cAMP-PKA-CREB Signaling Pathway
Source: Biomolecules. 2026 Apr 12;16(4):570. doi: 10.3390/biom16040570 (PMC13114105; doi:10.3390/biom16040570)
Supplement: Supplementary file 1 [file biomolecules-16-00570-s001.zip › biomolecules-4188919-supp figs.pdf]

Supporting Material

Variecolactone, a natural PDE4 inhibitor from Marine-Derived *Talaromyces* sp. ZSD-1, alleviates amyloid-β accumulation and mtDNA dyshomeostasis via cAMP-PKA-CREB signaling pathway

Tingting Fu[1, #], Yujia Shi[1, #], Zhonglin Yang[1], Juan Zhou[1],Bo Yi[2], Dongxiao Jia[1], Ling Huang[1, *], Wandi Xiong[1, *], Ying Fu[1, *]

[1] Key Laboratory of Tropical Biological Resources of Ministry of Education, School of Pharmaceutical Sciences, Hainan University, Haikou, China; hy0211065@muhn.edu.cn (T Fu); 23221055000018@hainanu.edu.cn (Y Shi); yzl030501@163.com (Z Yang); juanzhou@hainanu.edu.cn (J Zhou); 15514070831@163.com (D Jia).

[2] Department of Pharmacy, 928th Hospital of PLA Joint Logistics Support Force, Haikou 571159, China; ddzj_yb@163.com (B Yi).

* Correspondence: fuying926@163.com (Y Fu); linghuang@hainanu.edu.cn (L Huang); xiongwandi@hainanu.edu.cn (W Xiong).

[#] Author contribute equally.

Contents

Section S1. Spectroscopic data of the PDE4 inhibitory compounds isolated from *Talaromyces* sp. ZSD-21 1
Section 2. Spectra of compounds 1 — 9 4
Figure S1. ^{1}H NMR spectrum (Chloroform-*d*) of **1** 4
Figure S2. ^{13}C NMR spectrum (Chloroform-*d*) of **1** 4
Figure S3. HRESIMS spectrum of **1** 5
Figure S4. ^{1}H NMR spectrum (Dimethyl Sulfoxide-d_6) of **2** 5
Figure S5. ^{13}C NMR spectrum (Dimethyl Sulfoxide-d_6) of **2** 6
Figure S6. HRESIMS spectrum of **2** 6
Figure S7. ^{1}H NMR spectrum (Acetone-d_6) of **3** 7
Figure S8. ^{13}C NMR spectrum (Acetone-d_6) of **3** 7
Figure S9. HRESIMS spectrum of **3** 8
Figure S10. ^{1}H NMR spectrum (Methanol-d_4) of **4** 8
Figure S11. ^{13}C NMR spectrum (Methanol-d_4) of **4** 9
Figure S12. HRESIMS spectrum of **4** 9
Figure S13. ^{1}H NMR spectrum (Dimethyl Sulfoxide-d_6) of **5** 10
Figure S14. ^{13}C NMR spectrum (Dimethyl Sulfoxide-d_6) of **5** 10
Figure S15. HRESIMS spectrum of **5** 11
Figure S16. ^{1}H NMR spectrum (Dimethyl Sulfoxide-d_6) of **6** 11
Figure S17. ^{13}C NMR spectrum (Dimethyl Sulfoxide-d_6) of **6** 12
Figure S18. HRESIMS spectrum of **6** 12
Figure S19. ^{1}H NMR spectrum (Dimethyl Sulfoxide-d_6) of **7** 113
Figure S20. ^{13}C NMR spectrum (Dimethyl Sulfoxide-d_6) of **7** 13
Figure S21. HRESIMS spectrum of **7** 14
Figure S22. ^{1}H NMR spectrum (Acetone-d_6) of **8** 14
Figure S23. ^{13}C NMR spectrum (Acetone-d_6) of **8** 15
Figure S24. HRESIMS spectrum of **8** 15
Figure S25. ^{1}H NMR spectrum (Dimethyl Sulfoxide-d_6) of **9** 16
Figure S26. ^{13}C NMR spectrum (Dimethyl Sulfoxide-d_6) of **9** 16
Figure S27. HRESIMS spectrum of **9** 17

Section S1. Spectroscopic data of the PDE4 inhibitory compounds isolated from *Talaromyces* sp. ZSD-21

1.1. Variecolactone (**1**)

White solid; ^{1}H NMR (400 MHz, Chloroform-*d*) δ 1.49 (1H, m, H-1a), 1.09 (1H, dd, *J* = 14.7, 1.6 Hz, H-1b), 2.78 (1H, m, H-2), 2.23 (1H, m, H-3), 2.23 (1H, m, H-4a), 2.13 (1H, m, H-4b), 3.59 (1H, dd, *J* = 10.6, 1.9 Hz, H-6), 6.97 (1H, m, H-8), 2.76 (1H, m, H-9a), 2.13 (1H, m, H-9b), 2.13 (1H, m, H-10), 1.98 (1H, m, H-12a), 0.99 (1H, dt, *J* = 13.8, 3.3 Hz, H-12b), 1.49 (2H, m, H_2-13), 1.49 (1H, m, H-15), 2.38 (1H, td, *J* = 11.0, 5.4 Hz, H-16), 1.98 (1H, m, H-17a), 1.36 (1H, m, H-17b), 1.49 (1H, m, H-18a), 1.24 (1H, m, H-18b), 4.71 (1H, s, H-21a), 4.63 (1H, s, H-21b), 1.70 (3H, s, H_3-22), 0.69 (3H, d, *J* = 7.5 Hz, H_3-23), 0.90 (3H, s, H_3-24), 0.86 (3H, s, H_3-25); ^{13}C NMR (100 MHz, Chloroform-*d*) δ 41.0 (C-1), 39.9 (C-2), 38.1 (C-3), 44.9 (C-4), 115.6 (C-5), 51.9 (C-6), 125.4 (C-7), 144.8 (C-8), 29.9 (C-9), 38.8 (C-10), 39.1 (C-11), 34.5 (C-12), 35.3 (C-13), 43.6 (C-14), 48.1 (C-15), 48.3 (C-16), 30.1 (C-17), 40.0 (C-18), 171.1 (C-19), 150.7 (C-20), 110.6 (C-21), 19.4 (C-22), 16.1 (C-23), 21.9 (C-24), 18.5 (C-25) [1]; HRESIMS m/z 383.2590 $[M-H]^-$ (calcd. for $C_{25}H_{35}O_3$, 383.2592).

1.2. 1, 3, 6-Trihydroxy-7-methyl-9, 10-anthracenedione (**2**)

Brown powder; ^{1}H NMR (400 MHz, Dimethyl Sulfoxide-*d*$_6$) δ 6.41 (1H, br s, H-2), 7.00 (1H, br s, H-4), 7.45 (1H , br s, H-5), 7.12 (1H, br s, H-7), 2.39 (3H, s, H_3-11); ^{13}C NMR (100 MHz, Dimethyl Sulfoxide-*d*$_6$) δ 167.3 (C-1), 107.4 (C-2), 164.8 (C-3), 107.9 (C-4), 134.9 (C-4a), 120.2 (C-5), 147.6 (C-6), 124.0 (C-7), 161.3 (C-8), 108.0 (C-8a), 188.1 (C-9), 113.7 (C-9a), 182.0 (C-10), 132.9 (C-10a), 21.5 (C-11) [2]; HRESIMS m/z 269.0456 $[M-H]^-$ (calcd. for $C_{15}H_9O_5$, 269.0455).

1.3. 3-de-O-methylsulochrin (**3**)

Yellow powder; ^{1}H NMR (400 MHz, Acetone-*d*$_6$) δ 6.19 (2H, br s, H-3, H-5), 2.18 (3H, s, H_3-7), 6.93 (1H, d, *J* = 2.3 Hz, H-3′), 6.62 (1H, d, *J* = 2.3 Hz, H-5′); ^{13}C NMR (100 MHz, Acetone-*d*$_6$) δ 110.8 (C-1), 162.5 (C-2), 108.7 (C-3), 148.0 (C-4), 108.7 (C-5), 162.5 (C-6), 21.9 (C-7), 200.9 (C-8), 125.9 (C-1′), 130.8 (C-2′), 108.5 (C-3′), 158.8 (C-4′), 107.3 (C-5′), 156.0 (C-6′), 167.0 (C-7′), 52.1 (C-8′) [3]; HRESIMS m/z 317.0666 $[M-H]^-$ (calcd. for $C_{16}H_{13}O_7$, 317.0667).

1.4. methyl 4-hydroxyphenylacetate (**4**)

Brown solid; ^{1}H NMR (400 MHz, Methanol-*d*$_4$) δ 6.77 (1H, d, *J* = 8.1 Hz, H-2), 7.12 (1H, d, *J* = 8.1 Hz, H-3), 7.12 (1H, d, *J* = 8.1 Hz, H-5), 6.77 (1H, d, *J* = 8.1 Hz, H-6), 3.57 (2H, s, H_2-7), 3.71 (3H, s, H_3-9); ^{13}C NMR (100 MHz, Methanol-*d*$_4$) δ 157.6 (C-1), 116.3 (C-2), 131.3 (C-3), 126.3 (C-4), 131.3 (C-5), 116.3 (C-6), 40.9 (C-7), 174.6 (C-8), 52.4 (C-9) [4]; HRESIMS m/z 165.0556 $[M-H]^-$ (calcd. for $C_9H_9O_3$, 165.0557).

1.5. 1-methyl-naphthalene-2, 6-dicarboxylic acid (**5**)

White powder; ^{1}H NMR (400 MHz, Dimethyl Sulfoxide-*d*$_6$) δ 7.79 (1H, d, *J* = 8.6 Hz, H-3), 8.02 (1H, d, *J* = 8.6 Hz, H-4), 8.60 (1H, s, H-6), 8.07 (1H, dd, *J* = 9.0, 1.4 Hz, H-8), 8.30 (1H, d, *J* = 9.0 Hz, H-9), 2.86 (3H, s, H_3-11); ^{13}C NMR (100 MHz, Dimethyl Sulfoxide-*d*$_6$) δ 134.8 (C-1), 131.5 (C-2), 126.2 (C-3), 127.4 (C-4), 133.1 (C-5), 130.7 (C-6), 129.1 (C-7), 125.8 (C-8), 125.7 (C-9), 134.3 (C-10), 15.6 (C-11), 169.8 (C-12), 167.2 (C-13) [5]; HRESIMS m/z 229.0510 $[M-H]^-$ (calcd. for $C_{13}H_9O_4$, 229.0506).

1.6. Chrysoxanthones C (**6**)

Yellow powder; ^{1}H NMR (400 MHz, Dimethyl Sulfoxide-*d*$_6$) δ 11.62 (1H, s, 1-OH), 7.44 (1H, d, *J* = 8.6, H-3), 6.66 (1H, d, *J* = 8.6, H-4), 3.81 (1H, dd, *J* = 11.0, 5.8 Hz, H-5), 2.89 (1H, m,

H-6), 2.65 (1H, m, H-7a), 2.45 (1H, m, H-7b), 13.63 (1H, s, 8-OH), 1.04 (3H, d, *J* = 6.1 Hz, H_3-11), 3.61 (3H, s, H_3-13), 3.60 (1H, d, *J* = 17.0 Hz, H-3a′), 3.12 (1H, d, *J* = 17.0 Hz, H-3b′), 11.84 (1H, s, 5′-OH), 7.50 (1H, d, *J* = 8.6 Hz, H-7′), 6.63 (1H, d, *J* = 8.6 Hz, H-8′), 4.60 (1H, d, *J* = 3.4 Hz, H-9′), 2.30 (1H, m, H-10′), 2.88 (1H, m, H-11a′), 2.28 (1H, m, H-11b′), 1.17 (3H, d, *J* = 5.8 Hz, H_3-13′), 3.71 (3H, s, H_3-15′); ^{13}C NMR (100 MHz, Dimethyl Sulfoxide-d_6) δ 159.0 (C-1), 117.3 (C-2), 140.1 (C-3), 107.5 (C-4), 158.2 (C-4a), 75.2 (C-5), 29.3 (C-6), 35.9 (C-7), 178.3 (C-8), 101.7 (C-8a), 186.4 (C-9), 106.3 (C-9a), 85.2 (C-10a), 17.7 (C-11), 170.0 (C-12), 52.8 (C-12), 84.4 (C-2′), 38.7 (C-3′), 195.7 (C-4′), 107.5 (C-4a′), 158.6 (C-5′), 118.1 (C-6′), 140.7 (C-7′), 107.2 (C-8′), 158.5 (C-8a′), 86.6 (C-9′), 29.9 (C-10′), 35.7 (C-11′), 175.7 (C-12′), 19.9 (C-13′), 168.9 (C-14′), 53.5 (C-15′) [6]; HRESIMS m/z 661.1529 $[M+Na]^+$ (calcd. for $C_{32}H_{30}O_{14}Na$, 661.1528).

1.7. Secalonic acid A (**7**)

Yellow powder; ^{1}H NMR (400 MHz, Dimethyl Sulfoxide-d_6) δ 11.65 (1H, s, 1-OH), 7.46 (1H, d, *J* = 8.6, H-3), 6.62 (1H, d, *J* = 8.6, H-4), 3.81 (1H, dd, *J* = 11.2, 5.8 Hz, H-5), 2.31 (1H, m, H-6), 2.68 (1H, d, *J* = 6.0 Hz, H-7), 2.64 (1H, d, *J* = 6.0 Hz, H-7), 13.60 (1H, s, 8-OH), 1.40 (3H, d, *J* = 6.4 Hz, H_3-11), 3.61 (3H, s, H_3-13); ^{13}C NMR (100 MHz, Dimethyl Sulfoxide-d_6) δ 158.9 (C-1), 117.3 (C-2), 140.2 (C-3), 107.5 (C-4), 158.6 (C-4a), 75.2 (C-5), 30.4 (C-6), 35.9 (C-7), 178.3 (C-8), 101.6 (C-8a), 186.6 (C-9), 106.3 (C-9a), 85.2 (C-10a), 17.7 (C-11), 170.0 (C-12), 52.8 (C-12) [7]; HRESIMS m/z 639.1705 $[M+H]^+$ (calcd. for $C_{32}H_{31}O_{14}$, 639.1708) and m/z 661.1524 $[M+Na]^+$ (calcd. for $C_{32}H_{30}O_{14}Na$, 661.1528).

1.8. Secalonic acid F (**8**)

Yellow powder; ^{1}H NMR (400 MHz, Acetone-d_6) δ 11.64 (1H, s, 1-OH), 7.49 (1H, s, H-3), 6.58 (1H , s, H-4), 3.97 (1H, m, H-5), 2.47 (1H, m, H-6), 2.84 (2H, m, H_2-7), 13.77 (1H, s, 8-OH), 1.16 (3H, d, *J* = 6.4 Hz, H_3-11), 3.64 (3H, s, H_3-13); ^{13}C NMR (100 MHz, Acetone-d_6) δ 160.2 (C-1), 118.6 (C-2), 141.2 (C-3), 108.4 (C-4), 160.2 (C-4a), 77.8 (C-5), 30.8 (C-6), 36.8 (C-7), 179.5 (C-8), 102.9 (C-8a), 188.6 (C-9), 107.5 (C-9a), 86.2 (C-10a), 18.3 (C-11), 171.1 (C-12), 53.0 (C-13) [8]; HRESIMS m/z 639.1707 $[M+H]^+$ (calcd. for $C_{32}H_{31}O_{14}$, 639.1708) and m/z 661.1526 $[M+Na]^+$ (calcd. for $C_{32}H_{30}O_{14}Na$, 661.1528).

1.9. RF-3192C (**9**)

Yellow oil; ^{1}H NMR (400 MHz, Dimethyl Sulfoxide-d_6) δ 5.89 (1H, s, H-2), 7.50 (1H, d, *J* = 2.2 Hz, H-5), 10.68 (1H, s, 6-OH), 6.36 (1H, d, *J* = 2.2 Hz, H-7), 13.84 (1H, s, 8-OH), 3.42 (1H, dd, *J* = 16.6, 1.6 Hz, H-2a′), 3.24 (1H, d, *J* = 16.6 Hz, H-2b′), 8.11 (1H, s, 3′-OH), 7.12 (1H, d, *J* = 2.2 Hz, H-5′), 11.32 (1H, s, 6′-OH), 6.48 (1H, d, *J* = 2.2 Hz, H-7′), 12.55 (1H, s, 8′-OH); ^{13}C NMR (100 MHz, Dimethyl Sulfoxide-d_6) δ 189.8 (C-1), 98.8 (C-2), 171.5 (C-3), 121.6 (C-4), 130.4 (C-4a), 105.0 (C-5), 162.4 (C-6), 104.9 (C-7), 163.9 (C-8), 107.5 (C-8a), 198.6 (C-1′), 49.2 (C-2′), 112.1 (C-3′), 146.1 (C-4′), 134.7 (C-4a′), 109.0 (C-5′), 164.8 (C-6′), 104.7 (C-7′), 164.7 (C-8′), 109.1 (C-8a′) [9]; HRESIMS m/z 379. 0457 $[M-H]^-$ (calcd. for $C_{20}H_{11}O_8$, 379. 0459).

References

1. Fujimoto, H.; Nakamura, E.; Okuyama, E.; Ishibashi, M. Immunomodulatory Constituents from an Ascomycete, Emericella aurantio-brunnea. *Chem. Pharm. Bull.* **2000**, *48*, 1436-1441.
2. Shoupeng, Z.; Rong, H.; Fangfang, L.; Hongxia, W.; Xiaowei, F.; Xiaosong, X.; Dongguo, L.; Shaohua, W.; Jian, H. Antiviral anthraquinones and azaphilones produced by an endophytic fungus *Nigrospora* sp. from Aconitum carmichaeli. *Fitoterapia* **2016**, *112*, 85-89.
3. Lihua, Z.; Baomin, F.; Yuqing, Z.; Yi, S.; Bing, L.; Fang, L.; Gang, C.; Jiao, B.; Huiming, H.; Haifeng,

W.; Yuehu, P. Polyketide butenolide, diphenyl ether, and benzophenone derivatives from the fungus *Aspergillus flavipes* PJ03-11. *Bioorg. Med. Chem. Lett.* **2016**, *26*, 346-350.

4. Shuo, S.; Wei, L.; Jian, W. A novel and other bioactive secondary metabolites from a marine fungus *Penicillium oxalicum* 0312F1. *Nat. Prod. Res.* **2013**, *27*, 2286-2291.
5. Hao, G.; Qinghua, Z.; Miaomiao, J.; Jinshan, T.; Chengdu, M.; Kui, H.; Michio, N.; Naili, W.; Xinsheng, Y. Polyketides from a marine sponge-derived fungus *Mycelia sterilia* and proton-proton long-range coupling. *Magn. Reson. Chem.* **2008**, *46*, 1148-1152.
6. Tamam, E.E.; Mario, F.; Raja H. A.; Graf T. N.; Swanson S. M.; Falkinham J. O.; Wani M. C.; Pearce C. J.; Oberlies N. H. Biosynthetically Distinct Cytotoxic Polyketides from *Setophoma terrestris*. *Eur. J. Org. Chem.* **2015**, *2015*, 109-121.
7. Noinart, J.; Buttachon, S.; Dethoup, T.; Gales, L.; Pereira, J.A.; Urbatzka, R.; Freitas, S.; Lee, M.; Silva, A.M.S.; Pinto, M.M.M.; Vasconcelos, V.; Kijjoa, A. A New Ergosterol Analog, a New Bis-Anthraquinone and Anti-Obesity Activity of Anthraquinones from the Marine Sponge-Associated Fungus *Talaromyces stipitatus* KUFA 0207. *Mar. Drugs* **2017**, *15*, 139.
8. Natanong, Y.; Ratsami, L.; Watchara, S.; Tadanori, A.; Sophon, B. Secondary Metabolites and Their Biological Activity from *Aspergillus aculeatus* KKU-CT2. *Curr. Microbiol.* **2018**, *75*, 513-518.
9. Ngoc, N.T.; Vien, L.T.; Hanh, T.T.H.; Cuong, N.X.; Nam, N.H.; Minh, C.V. Chemical constituents of a marine-derived fungus *Aspergillus* sp. HL24 and their cytotoxic activity. *Tetrahedron* **2025**, *184*, 134778.

Section S2. Spectra of compounds 1 — 9

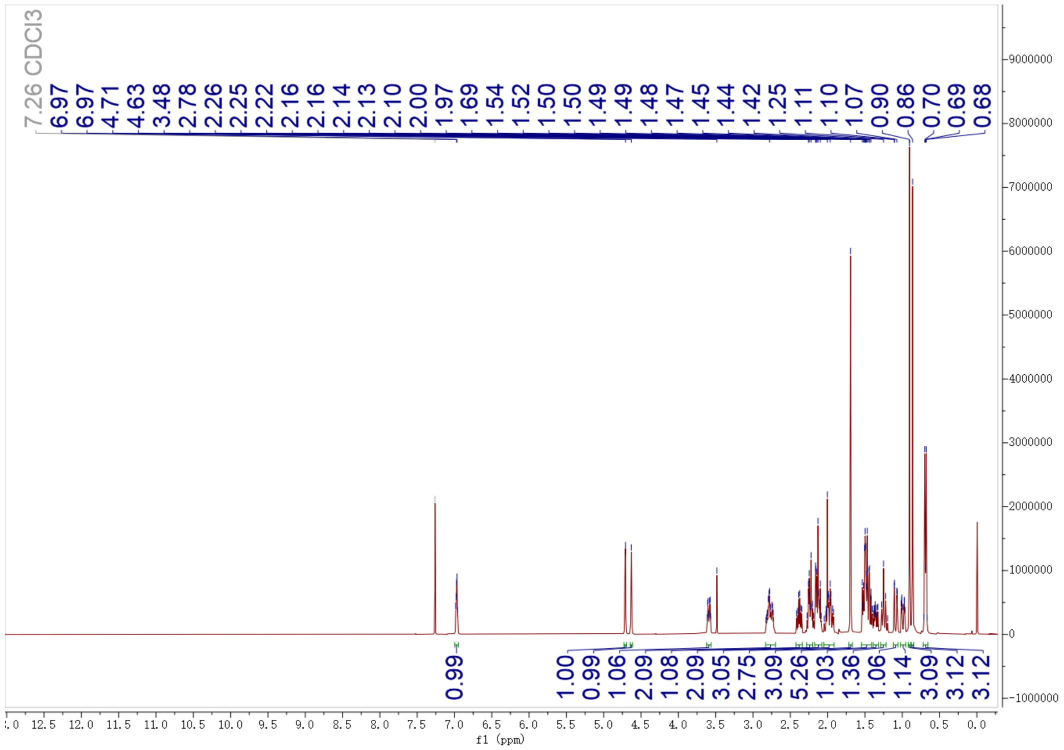

Figure S1. ^{1}H NMR spectrum (Chloroform-*d*) of **1**

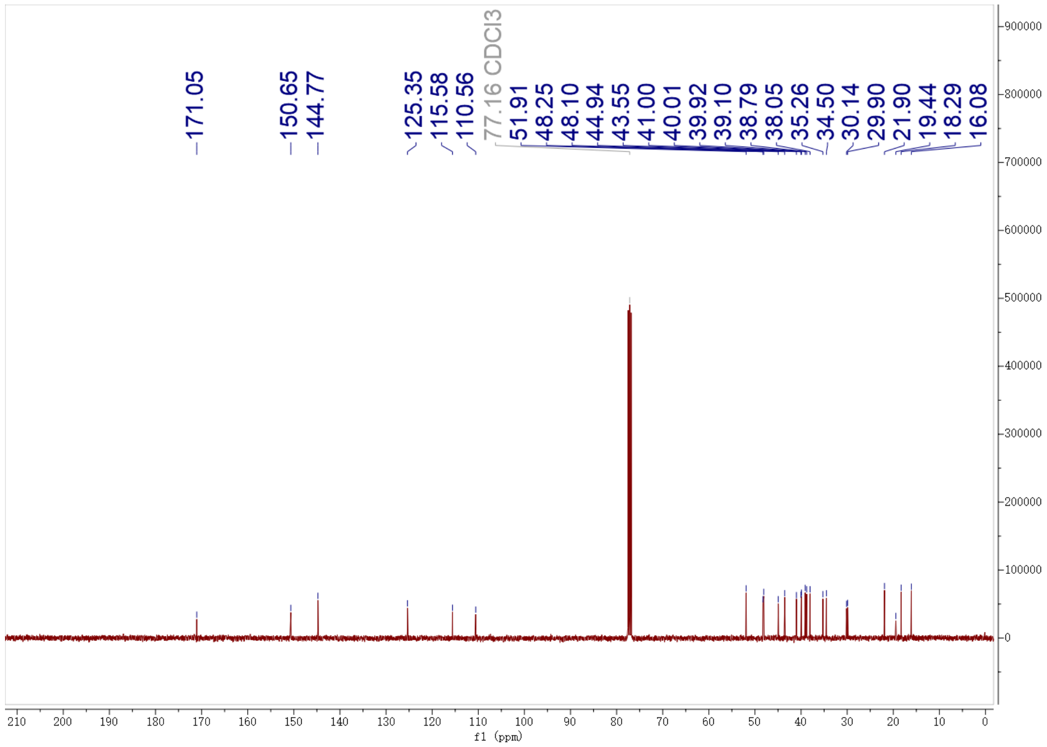

Figure S2. ^{13}C NMR spectrum (Chloroform-*d*) of **1**

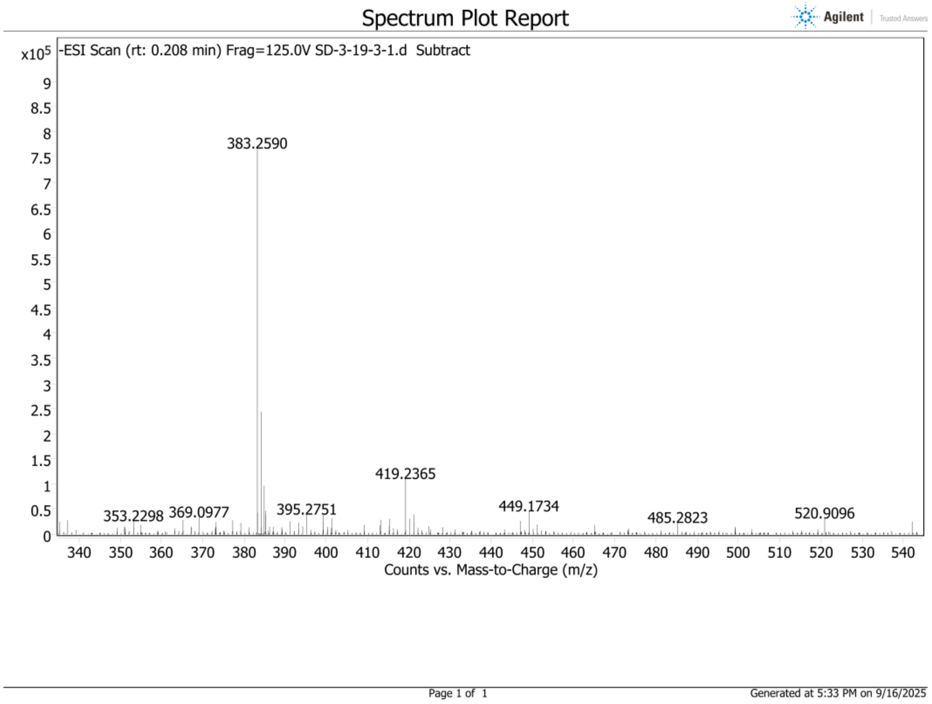

Figure S3. HRESIMS spectrum of **1**

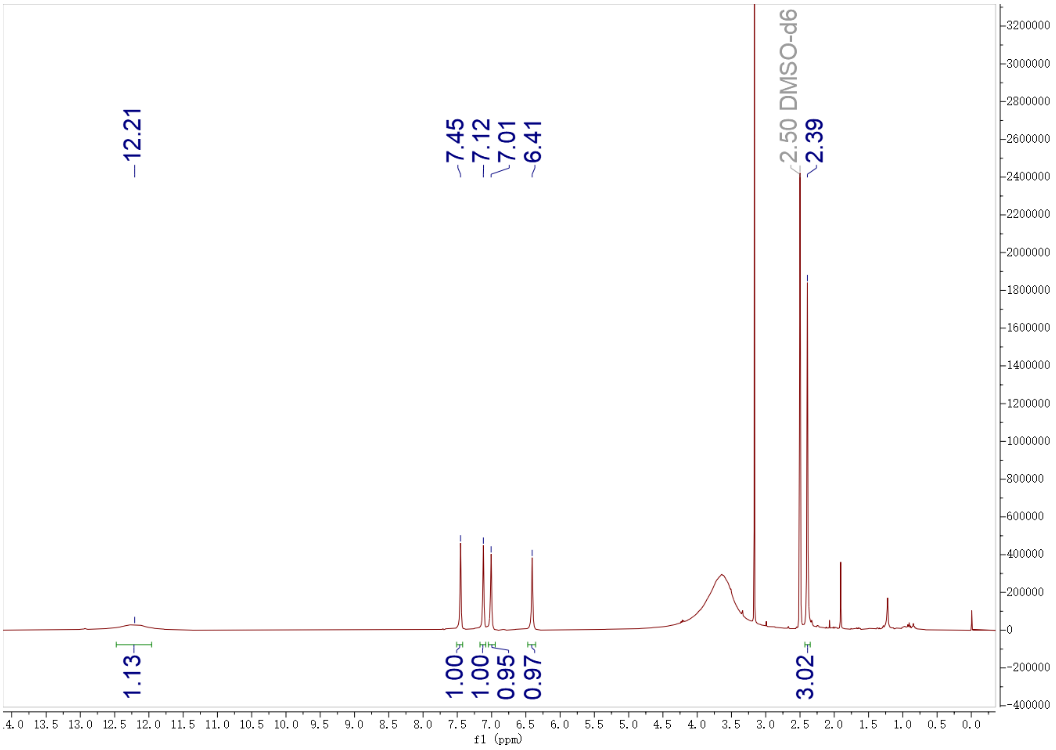

Figure S4. ^{1}H NMR spectrum (Dimethyl Sulfoxide-d_6) of **2**

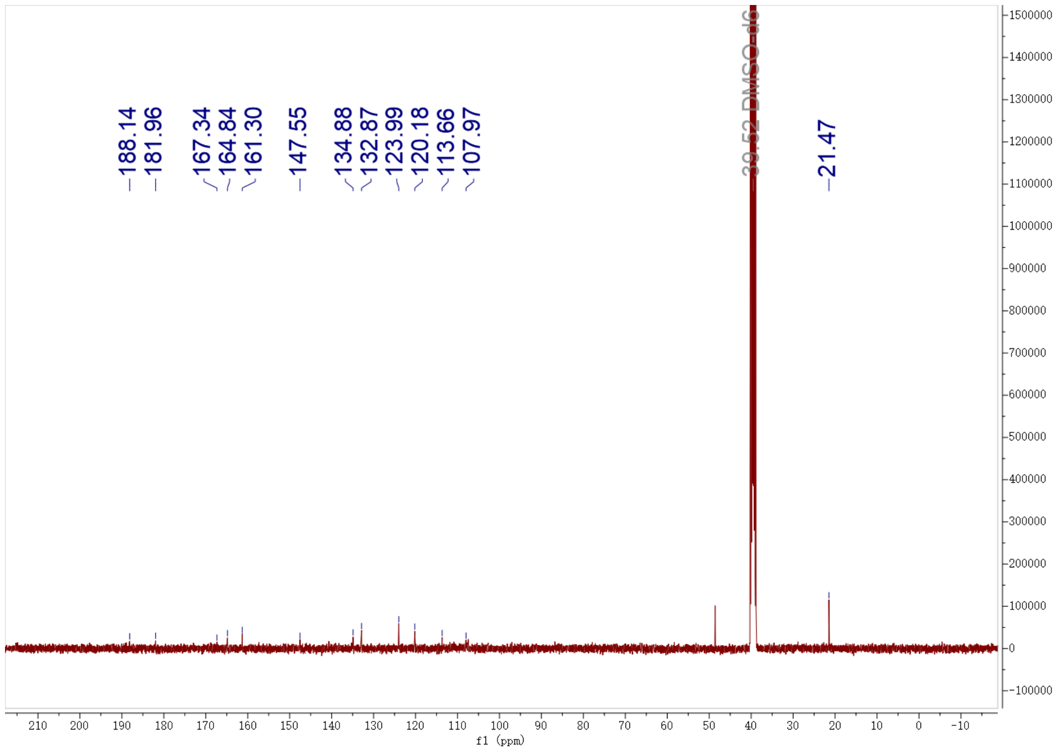

Figure S5. ^{13}C NMR spectrum (Dimethyl Sulfoxide-d_6) of **2**

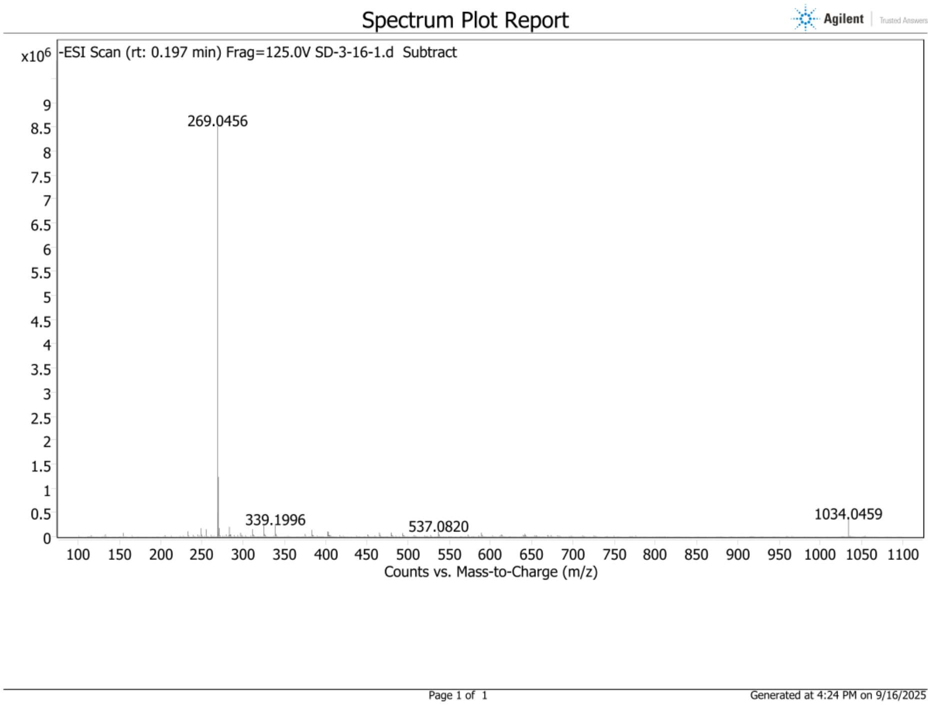

Figure S6. HRESIMS spectrum of 2

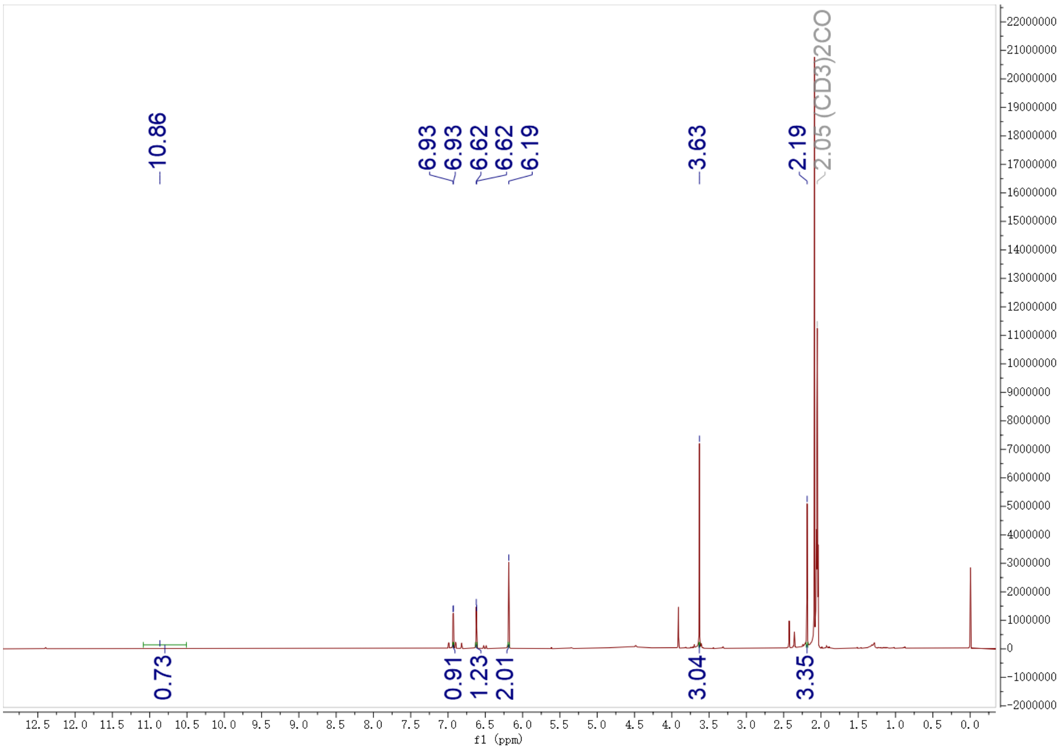

Figure S7. ^{1}H NMR spectrum (Acetone-d_6) of **3**

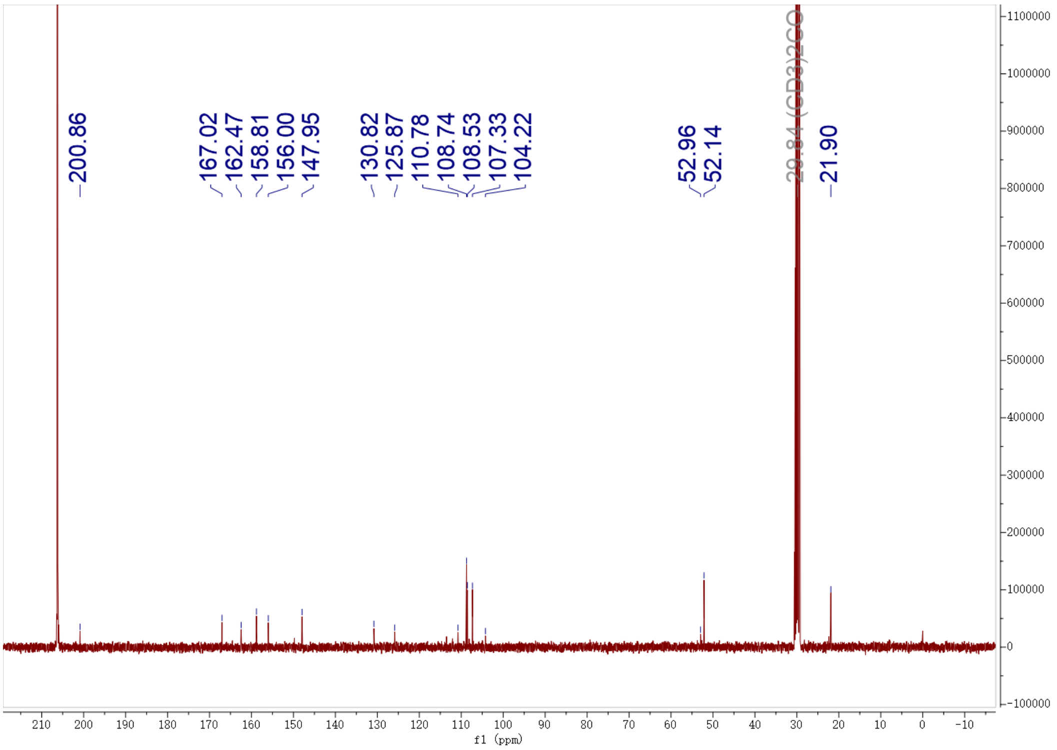

Figure S8. ^{13}C NMR spectrum (Acetone-d_6) of **3**

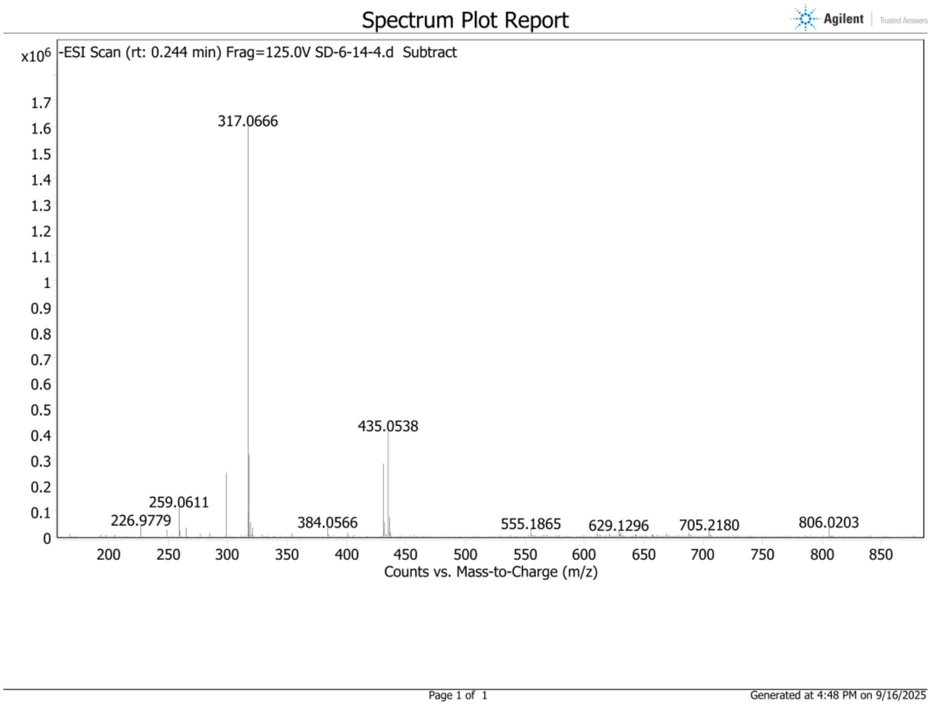

Figure S9. HRESIMS spectrum of **3**

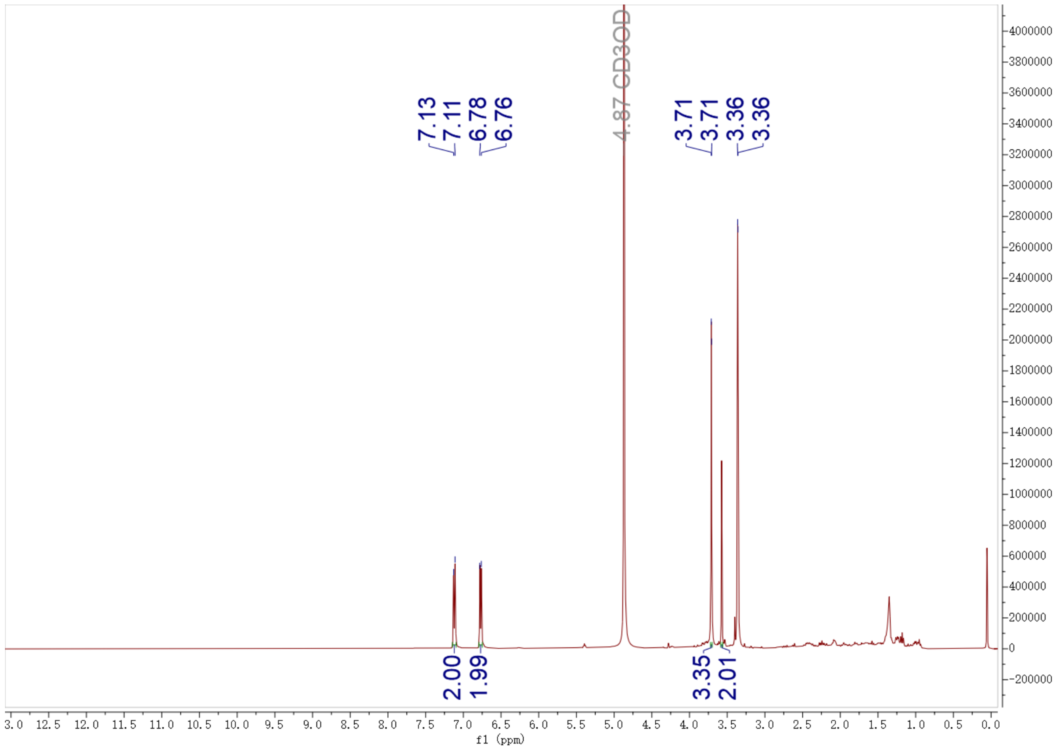

Figure S10. ^{1}H NMR spectrum (Methanol-d_4) of **4**

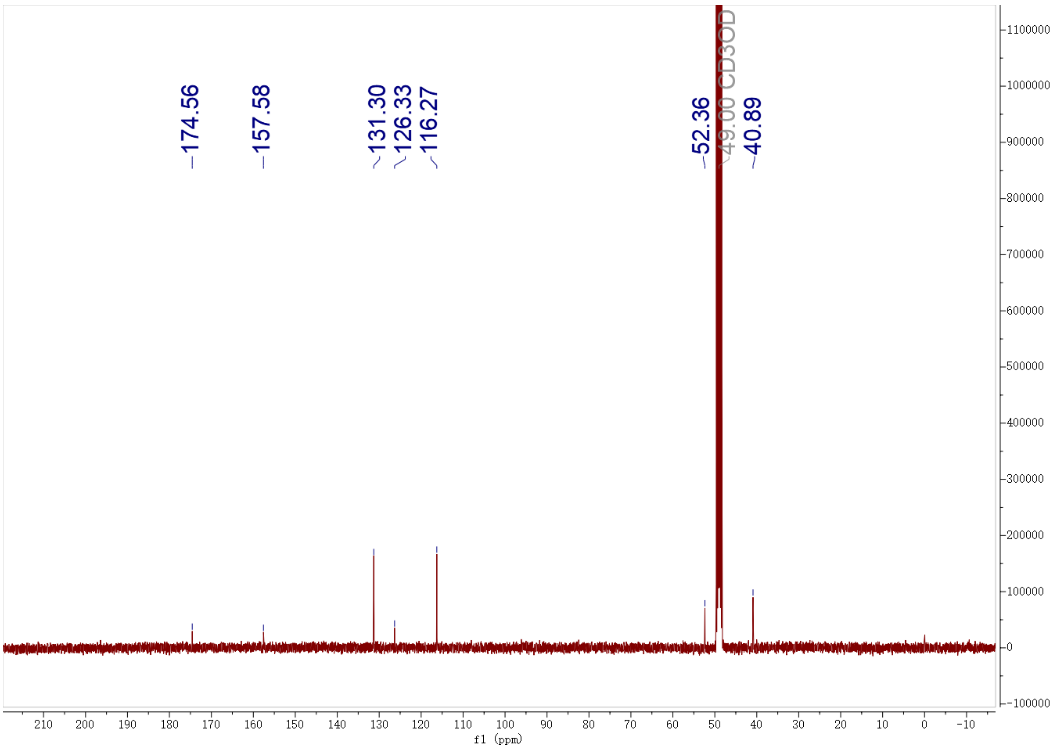

Figure S11. ^{13}C NMR spectrum (Methanol-d_4) of **4**

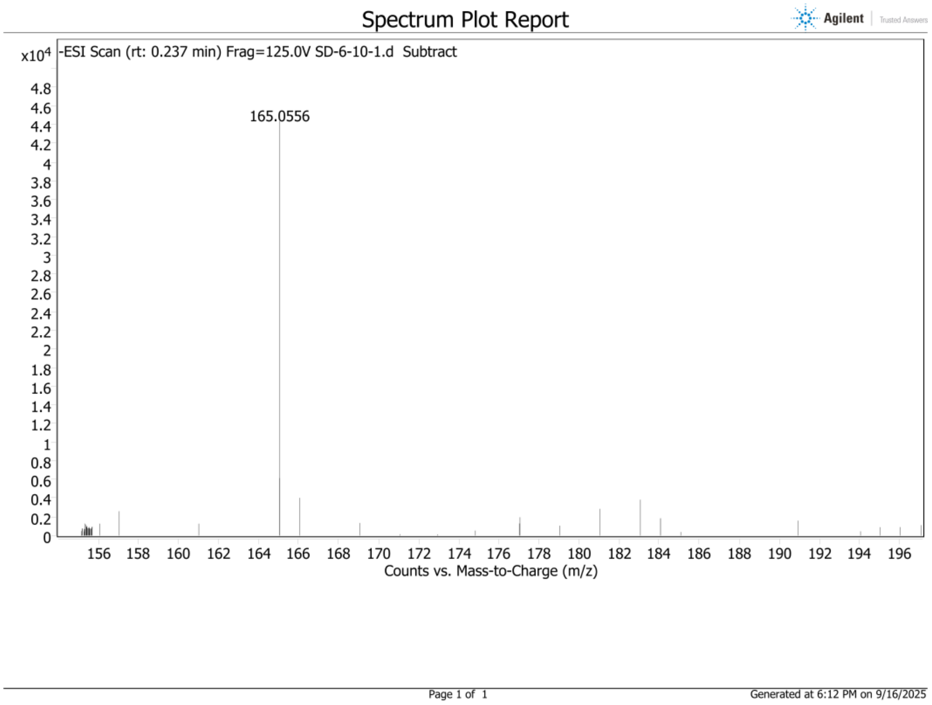

Figure S12. HRESIMS spectrum of **4**

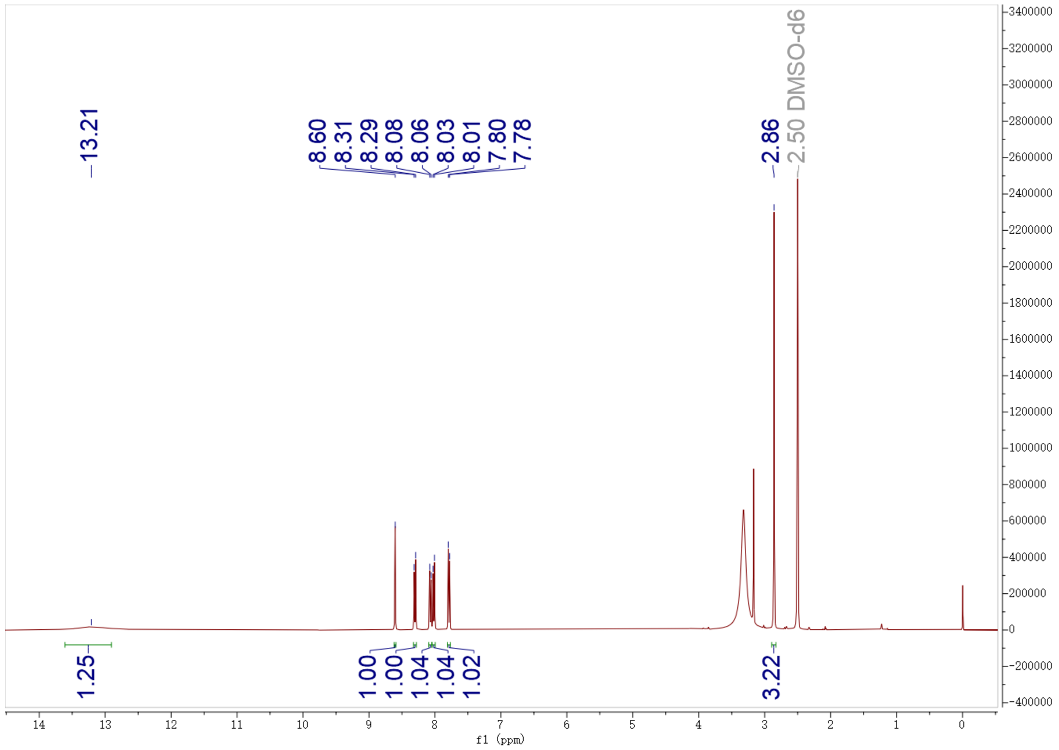

Figure S13. ^{1}H NMR spectrum (Dimethyl Sulfoxide-d_6) of **5**

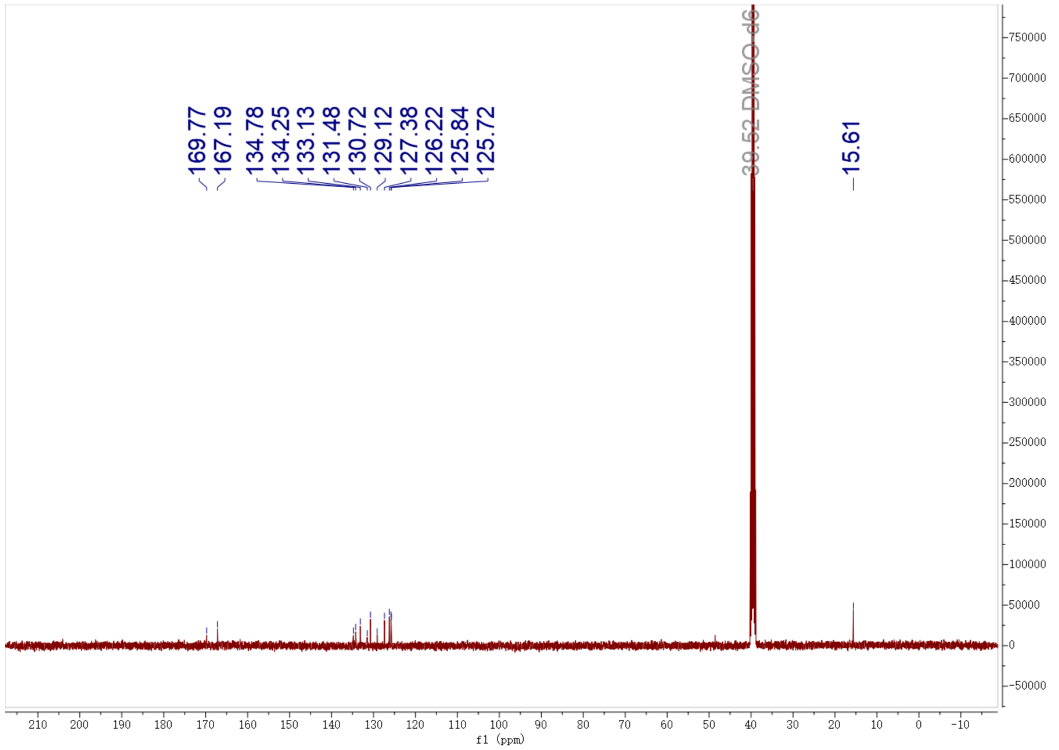

Figure S14. ^{13}C NMR spectrum (Dimethyl Sulfoxide-d_6) of **5**

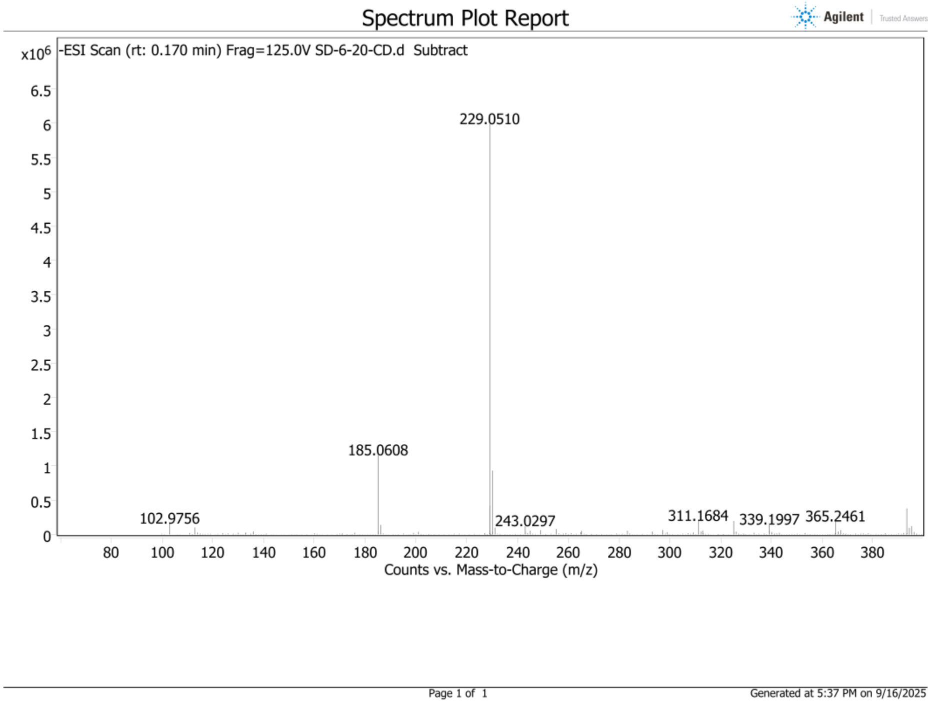

Figure S15. HRESIMS spectrum of **5**

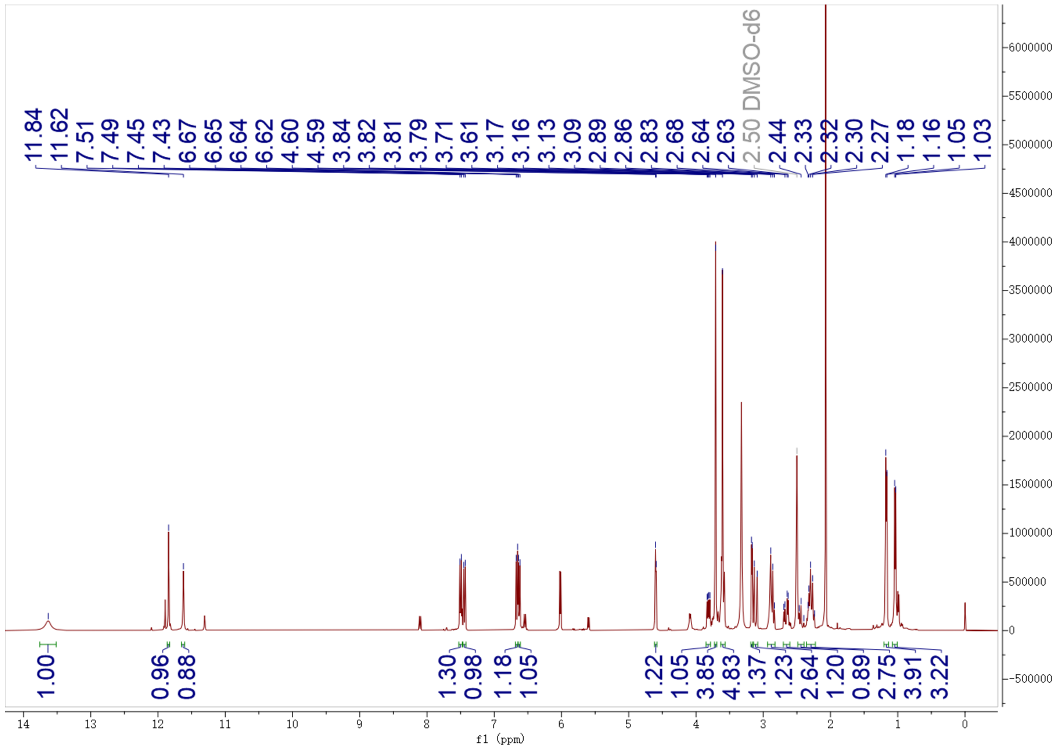

Figure S16. ^{1}H NMR spectrum (Dimethyl Sulfoxide-d_6) of **6**

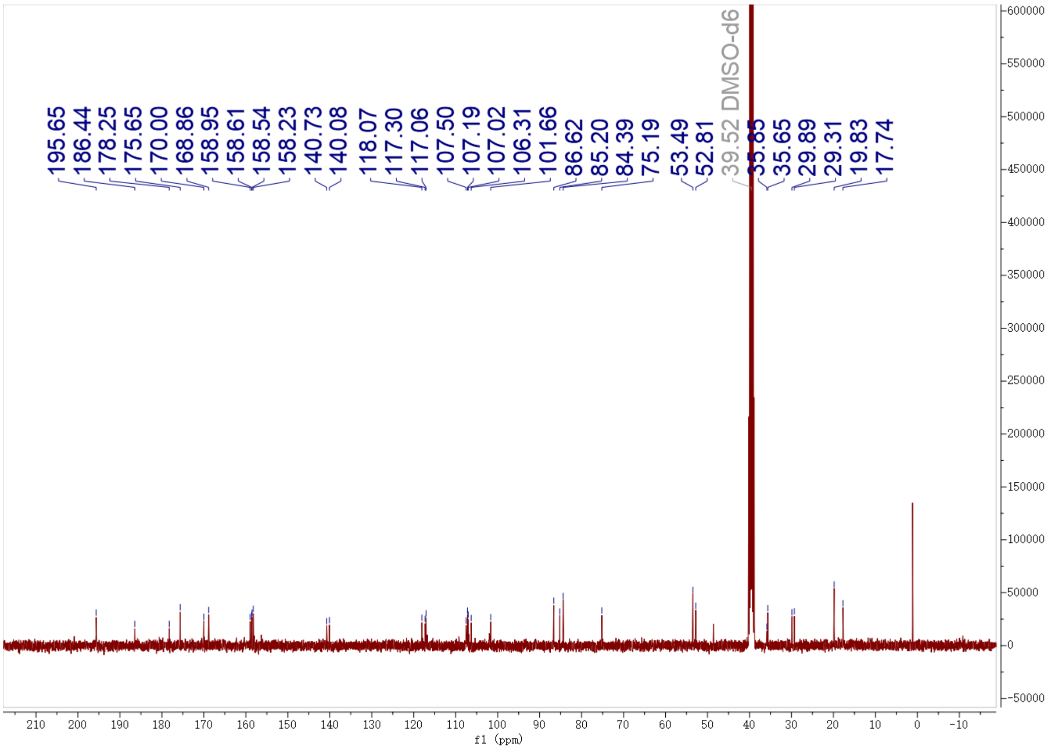

Figure S17. ^{13}C NMR spectrum (Dimethyl Sulfoxide-d_6) of **6**

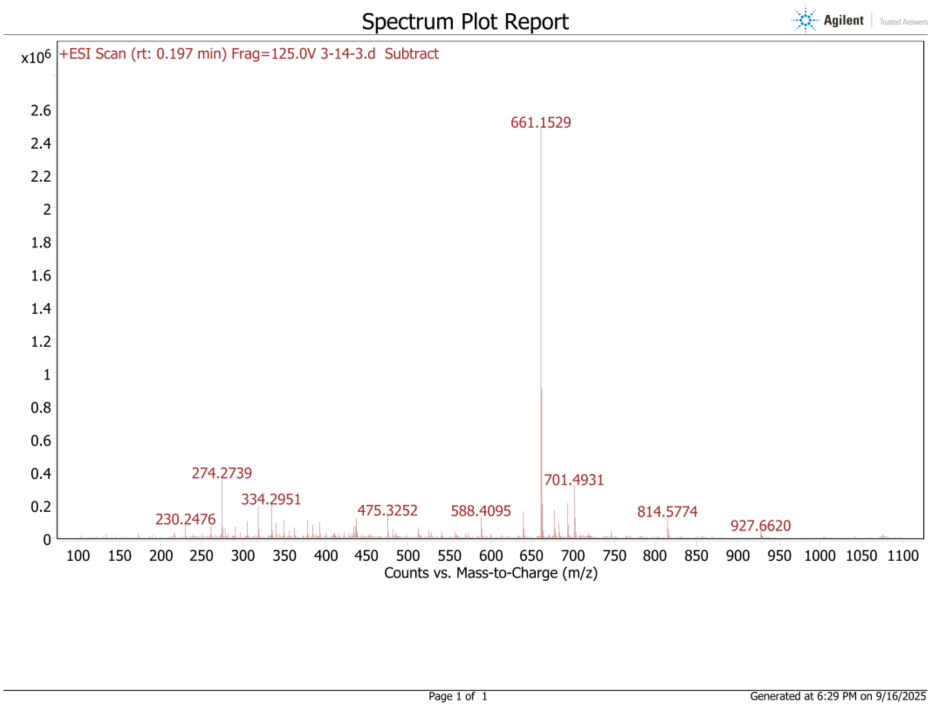

Figure S18. HRESIMS spectrum of **6**

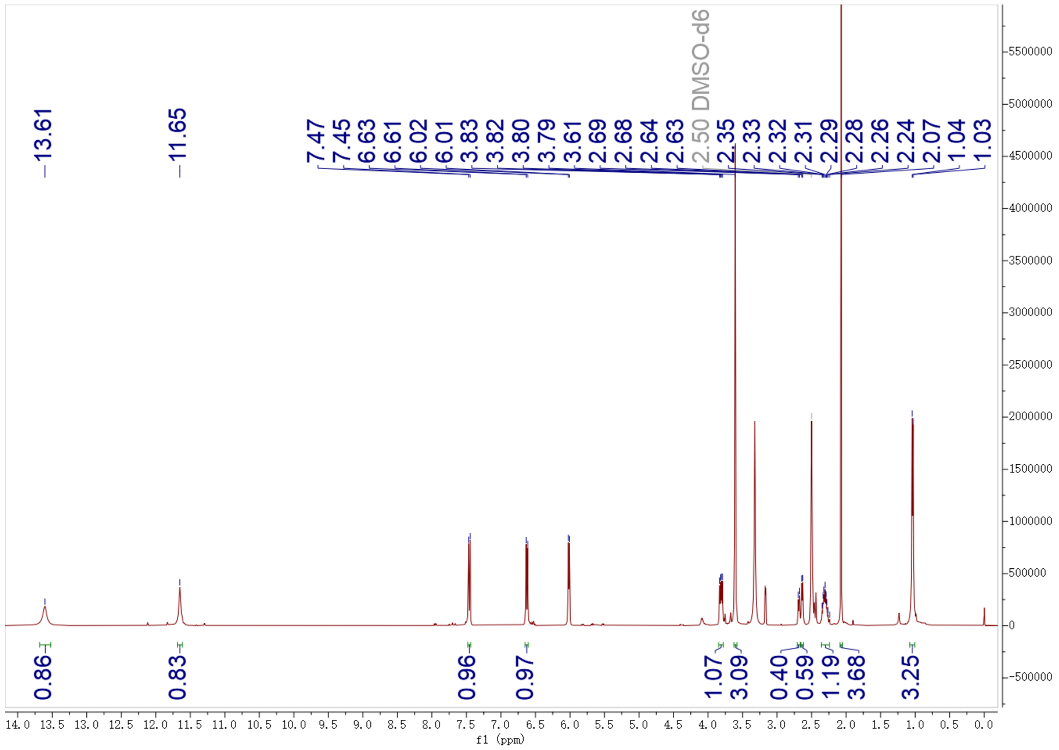

Figure S19. ^{1}H NMR spectrum (Dimethyl Sulfoxide-d_6) of **7**

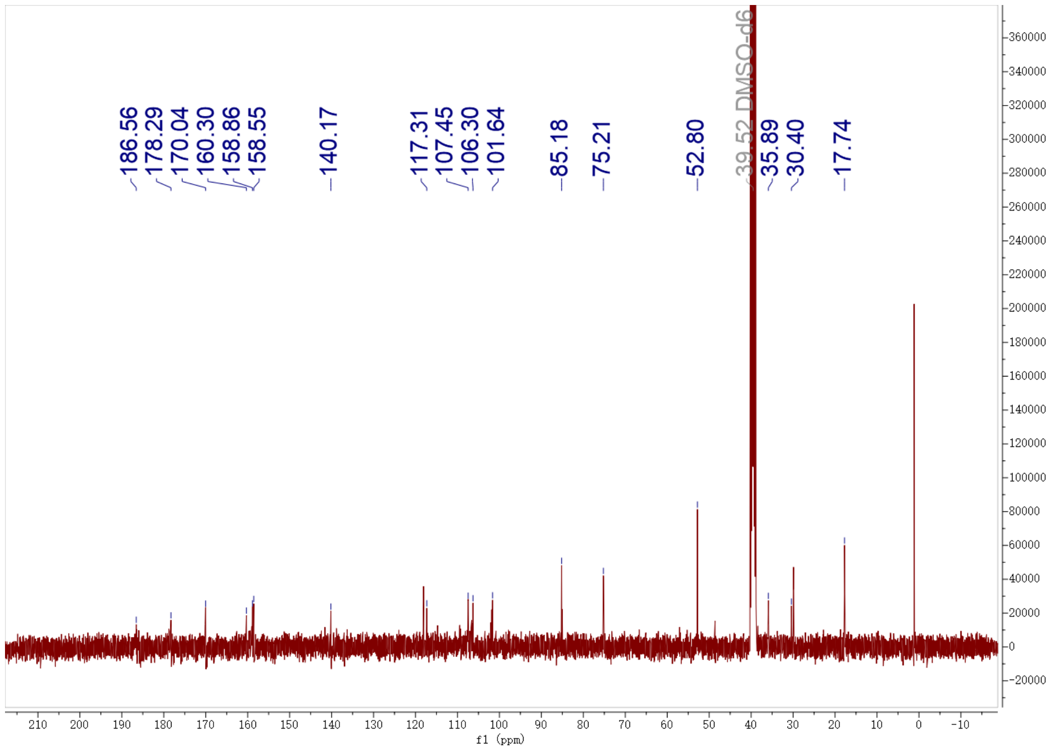

Figure S20. ^{13}C NMR spectrum (Dimethyl Sulfoxide-d_6) of **7**

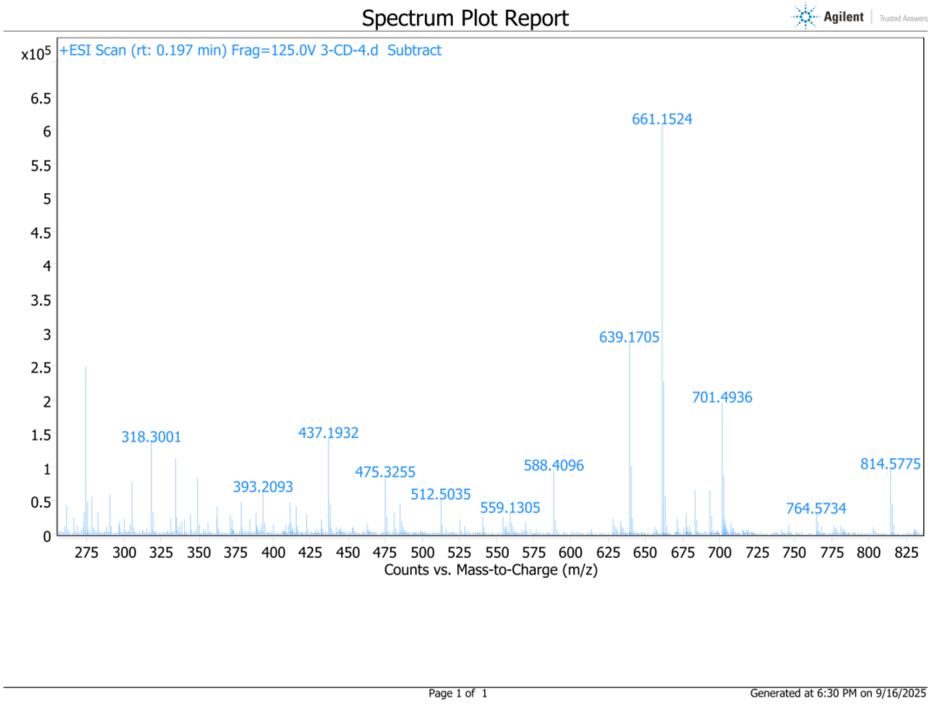

Figure S21. HRESIMS spectrum of **7**

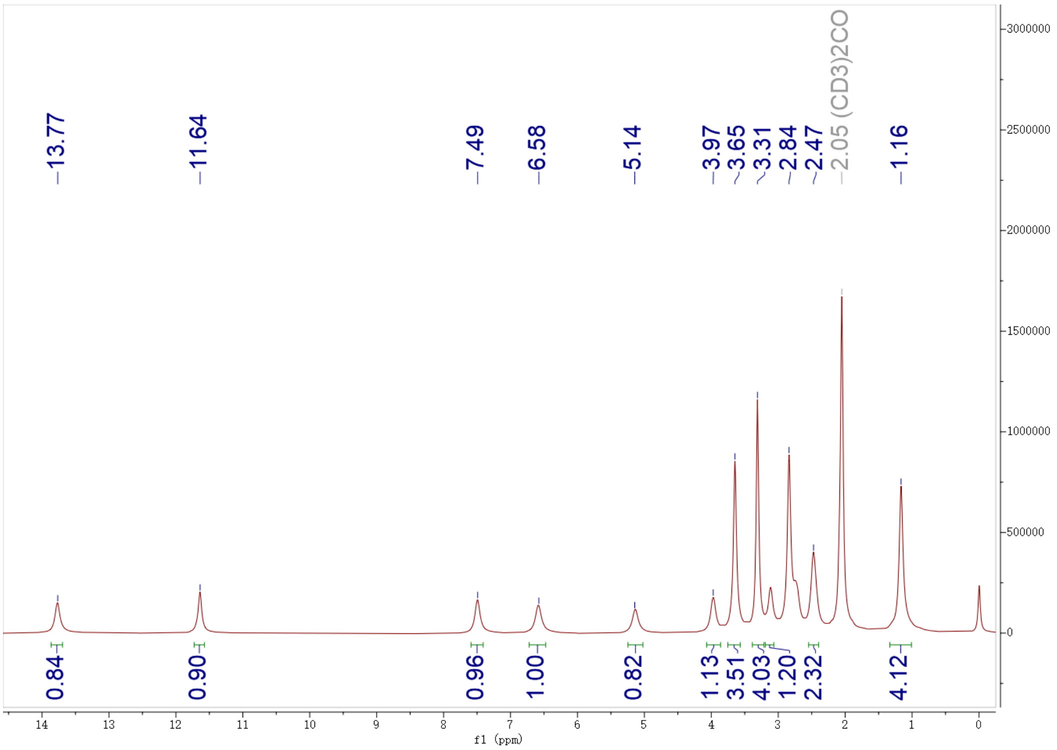

Figure S22. ^{1}H NMR spectrum (Acetone-d_6) of **8**

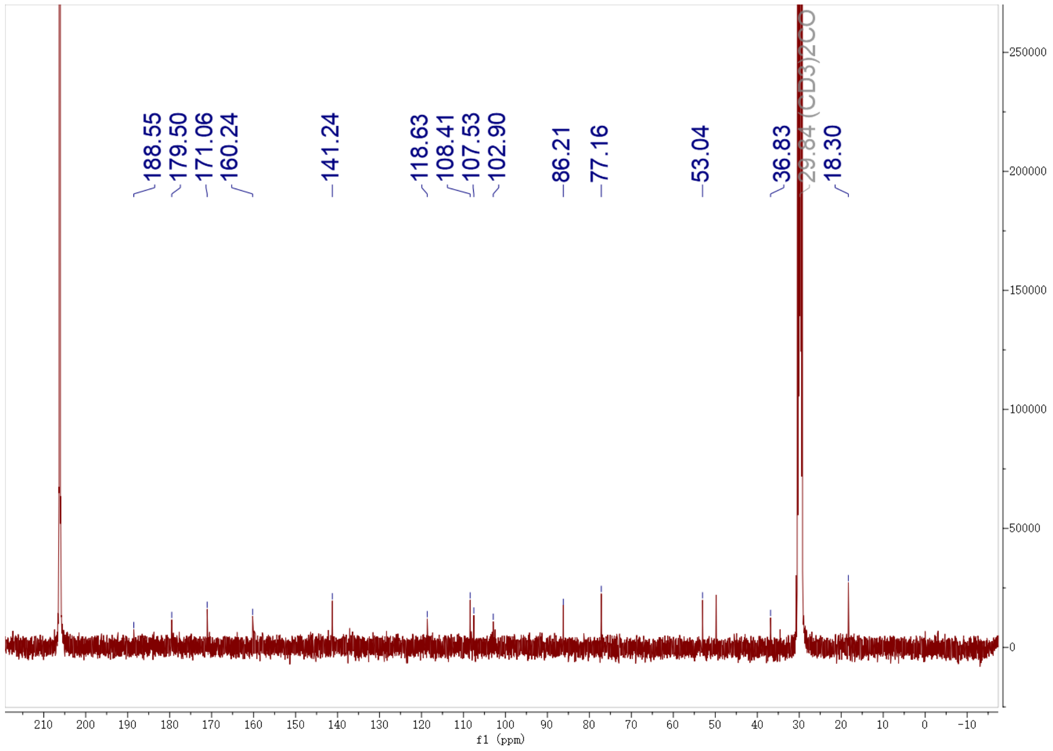

Figure S23. ^{13}C NMR spectrum (Acetone-d_6) of **8**

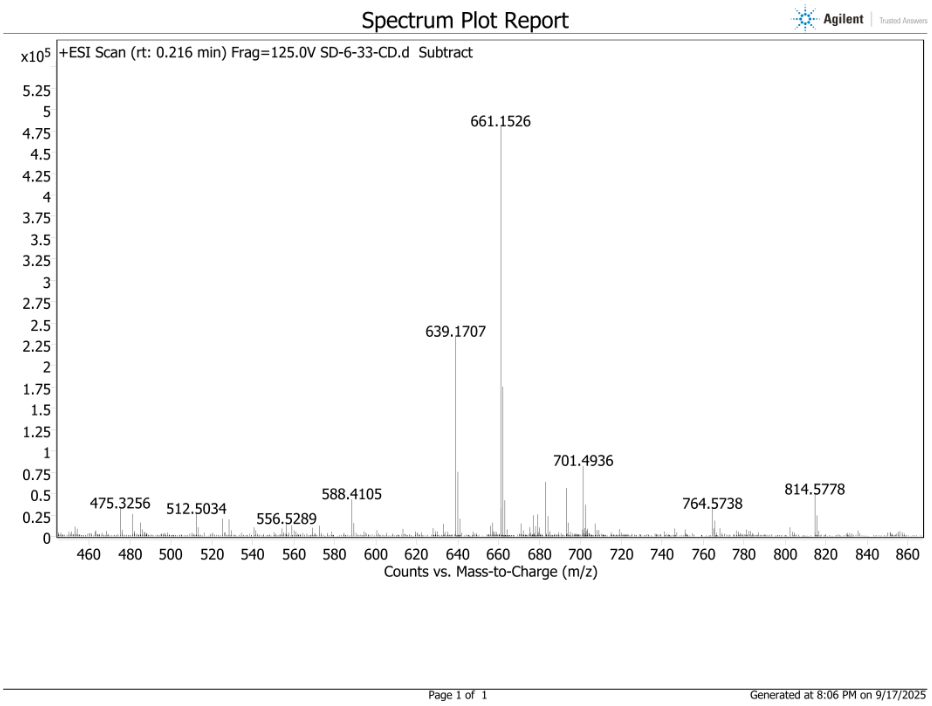

Figure S24. HRESIMS spectrum of **8**

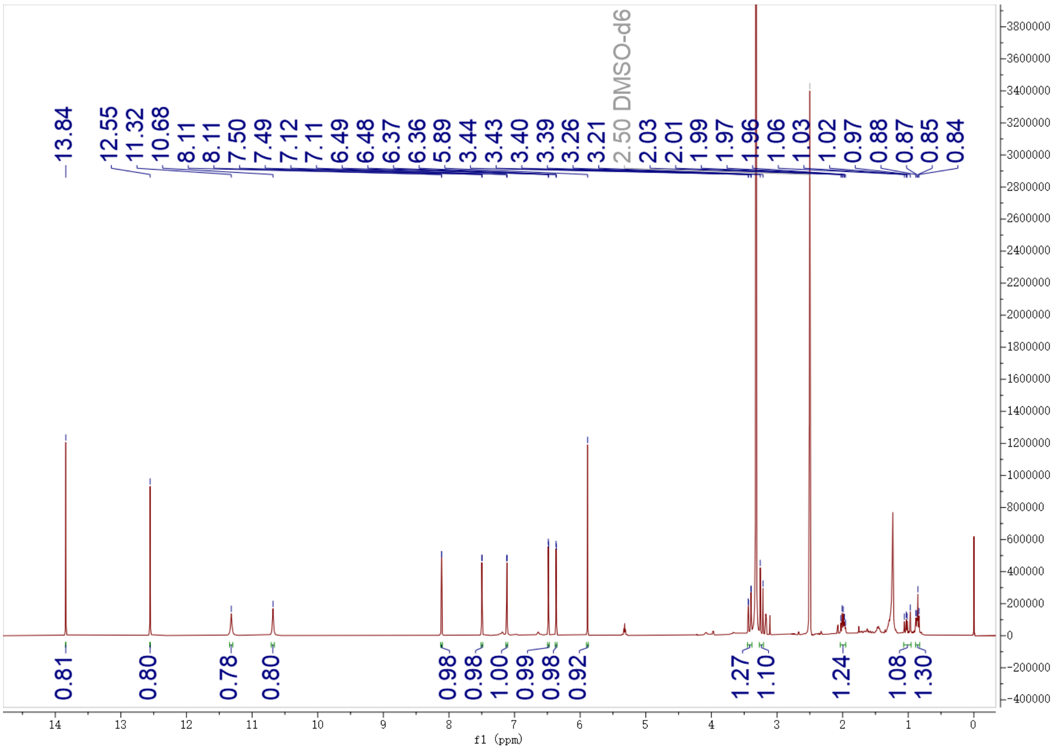

Figure S25. ^{1}H NMR spectrum (Dimethyl Sulfoxide-d_6) of **9**

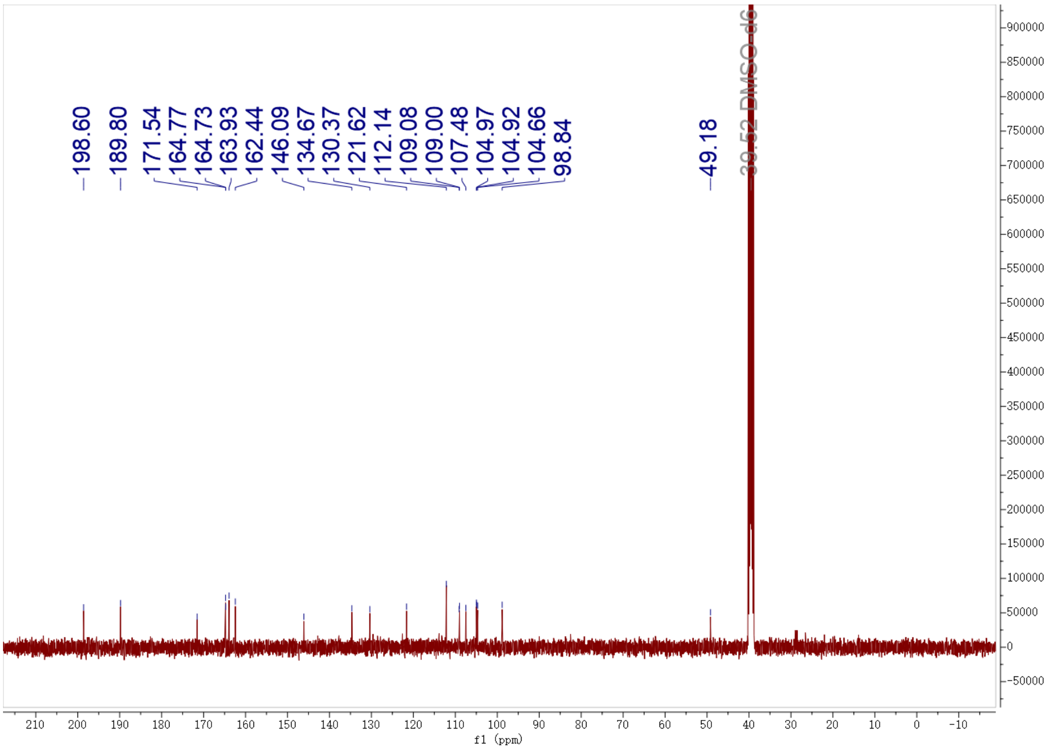

Figure S26. ^{13}C NMR spectrum (Dimethyl Sulfoxide-d_6) of **9**

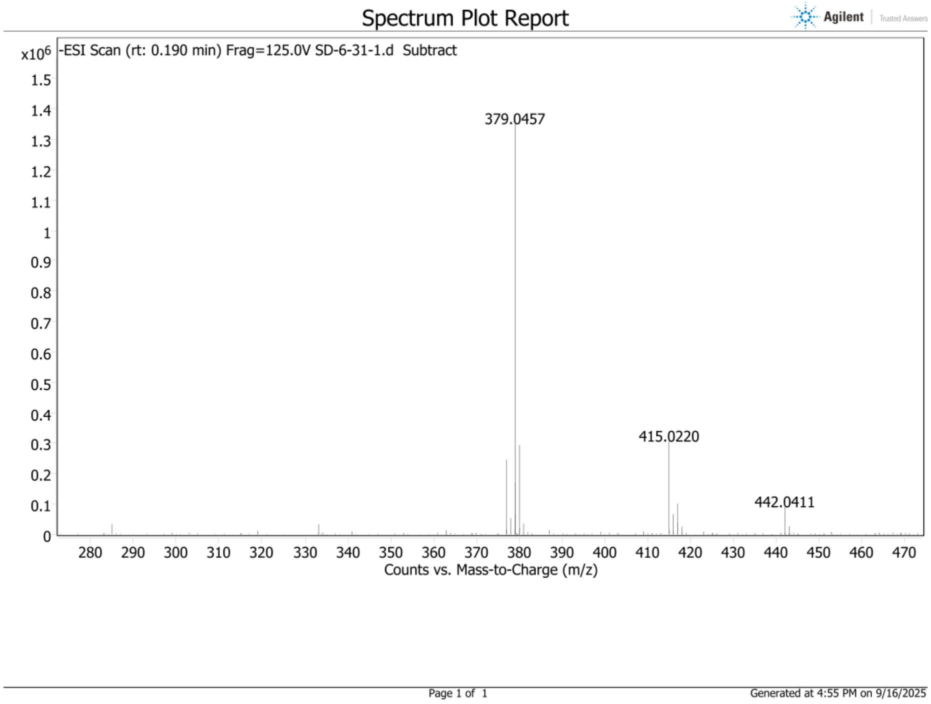

Figure S27. HRESIMS spectrum of **9**
