# Supplementary material for: Variecolactone, a Natural PDE4 Inhibitor from Marine-Derived Talaromyces sp. ZSD-1, Alleviates Amyloid-β Accumulation and mtDNA Dyshomeostasis via cAMP-PKA-CREB Signaling Pathway
Source: Biomolecules. 2026 Apr 12;16(4):570. doi: 10.3390/biom16040570 (PMC13114105; doi:10.3390/biom16040570)

Original WB figure

Figure 2M

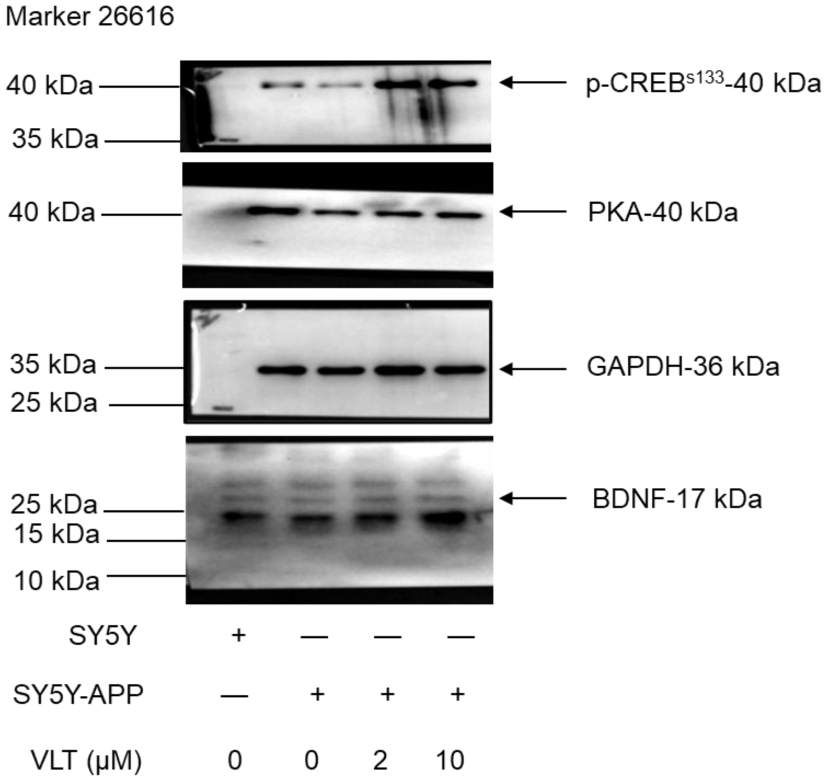

WB figure with clear marker in the repeated experiment

Figure 2M

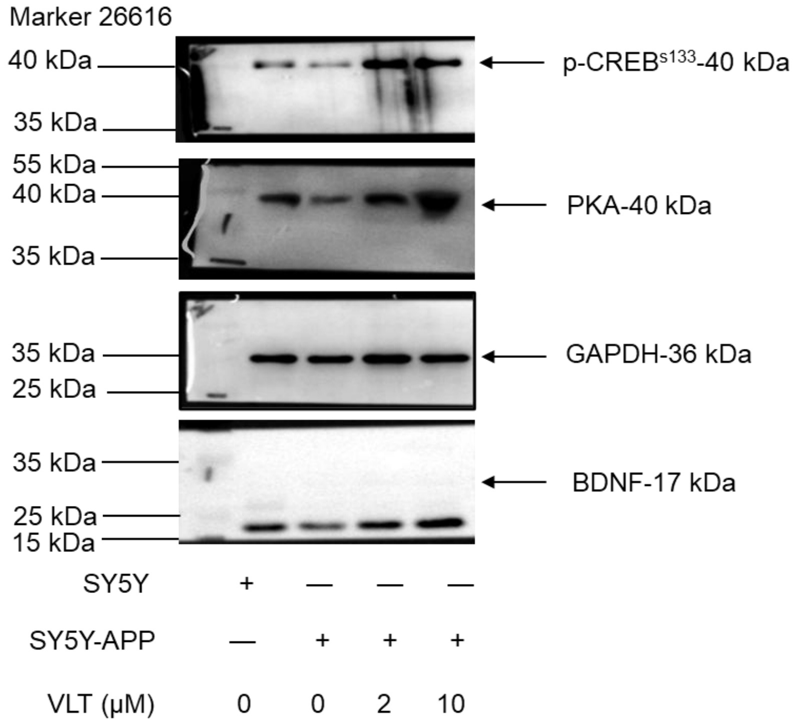

Original WB figure

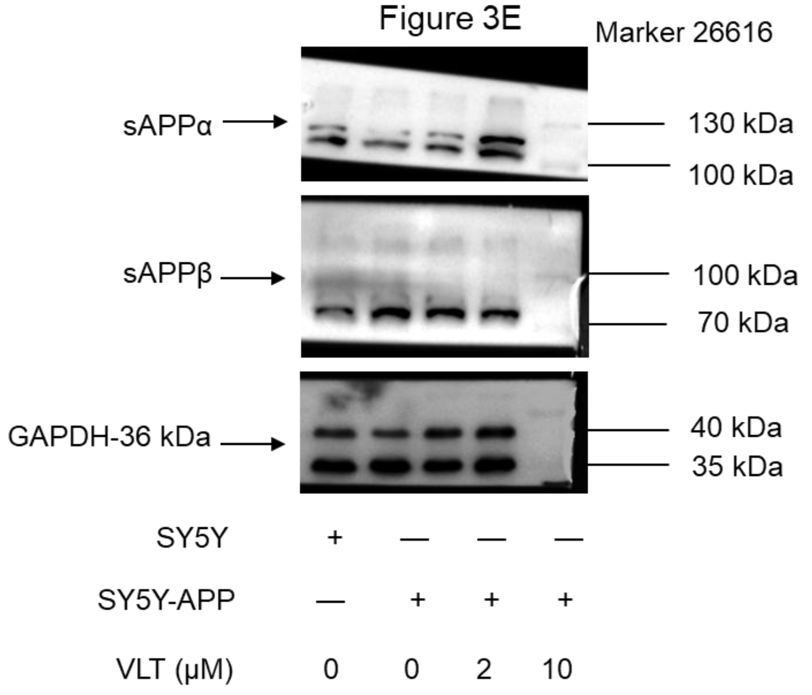

Original WB figure

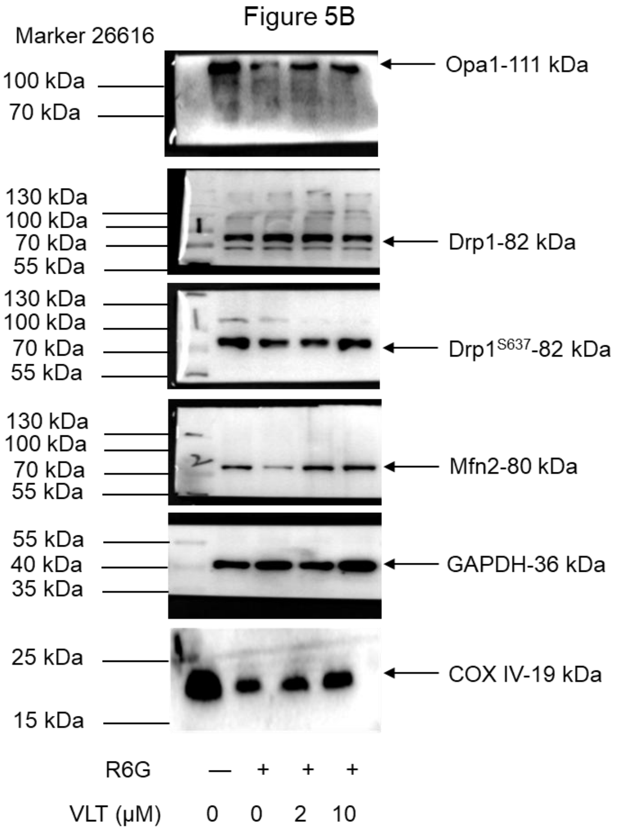

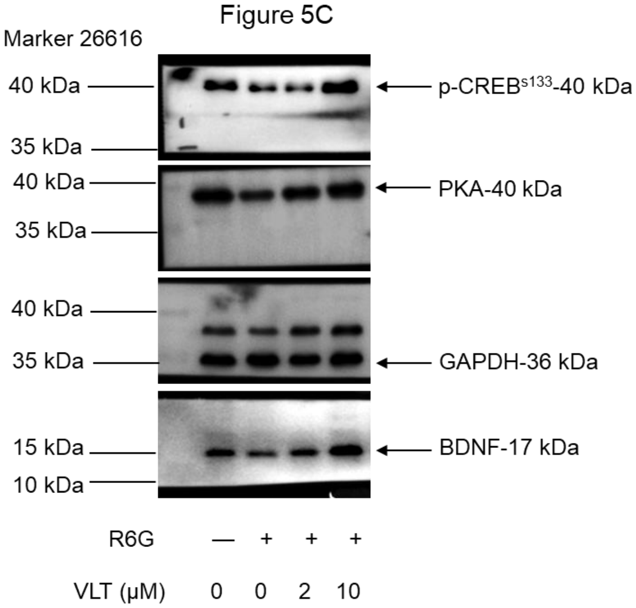

WB figure with clear marker in the repeated experiment

Figure 5B

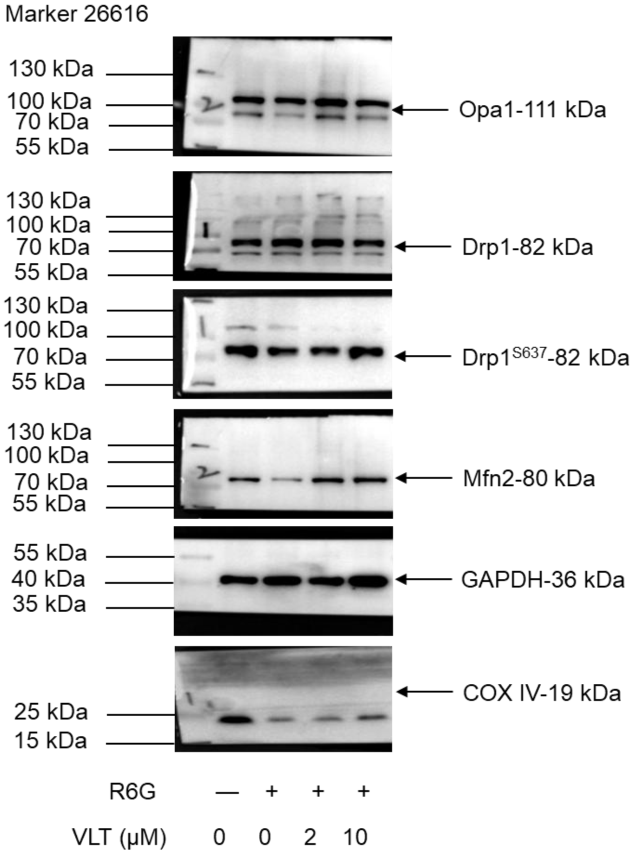

Figure 5C

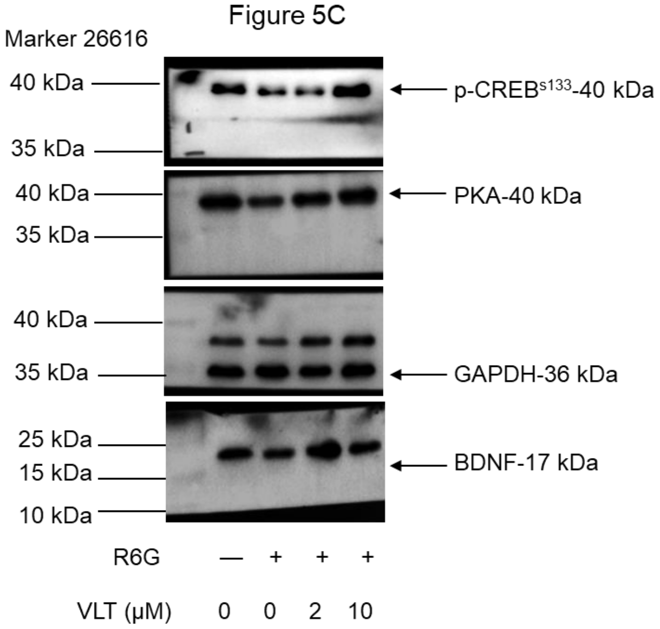

Original WB figure

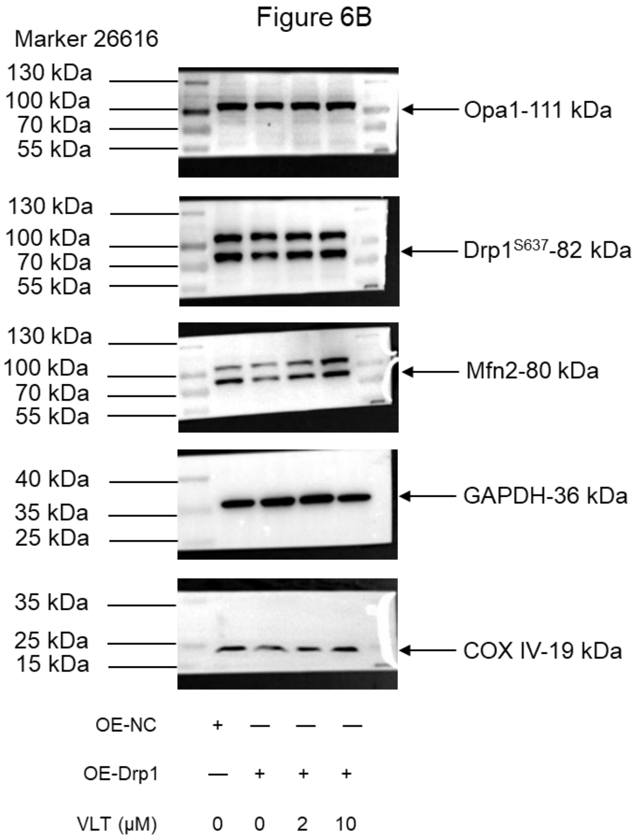

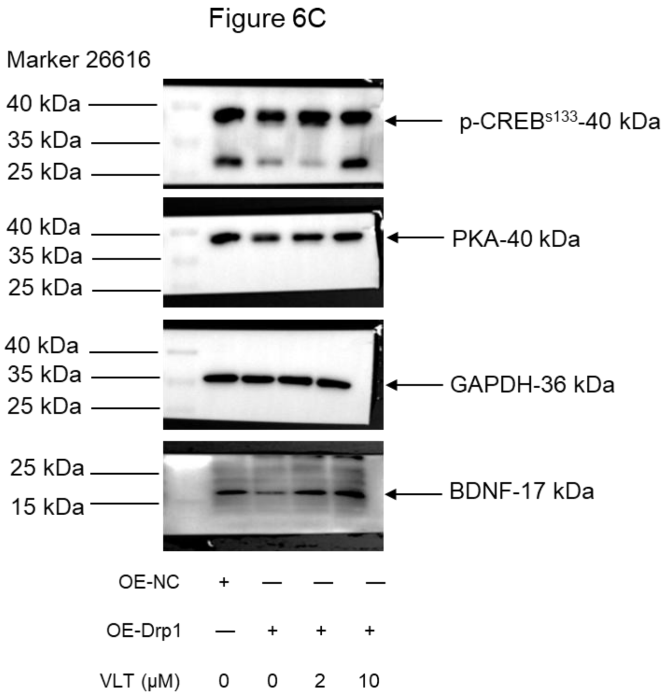

Supplement: Supplementary file 1 [file biomolecules-16-00570-s001.zip › biomolecules-4188919-supplementary.pdf]
